# Four New Flavonol Glycosides from the Leaves of *Brugmansia suaveolens*

**DOI:** 10.3390/molecules19056727

**Published:** 2014-05-22

**Authors:** Fabiana Geller, Renato Murillo, Lisa Steinhauser, Berta Heinzmann, Klaus Albert, Irmgard Merfort, Stefan Laufer

**Affiliations:** 1 Department of Pharmaceutical/Medicinal Chemistry, University of Tübingen, Tübingen 72076, Germany; 2 Escuela de Quimica and CIPRONA, University of Costa Rica, San José 2060, Costa Rica; 3 Department of Organic Chemistry, University of Tübingen, Tübingen 72076, Germany; 4 Department of Industrial Pharmacy, Federal University of Santa Maria, Santa Maria 97105-900, Brazil; 5 Department of Pharmaceutical Biology and Biotechnology, University of Freiburg, Freiburg im Breisgau 79104, Germany

**Keywords:** *Brugmansia suaveolens*, Solanaceae, acylated kaempferol glycosides

## Abstract

Four new flavonol glycosides were isolated from the leaves of *Brugmansia suaveolens*: kaempferol 3-*O*-β-d-glucopyranosyl-(1'''→2'')-*O*-α-L-arabinopyranoside (**1**), kaempferol 3-*O*-β-d-glucopyranosyl-(1'''→2'')-*O*-α-L-arabinopyranoside-7-*O*-į-d-gluco-pyranoside (**2**), kaempferol 3-*O*-β-d-[6'''-O-(E-caffeoyl)]-glucopyranosyl-(1'''→2'')-*O*-α-l-arabinopyranoside-7-*O*-β-d-glucopyranoside (**3**), and kaempferol 3-*O*-β-d-[2'''-O-(E-caffeoyl)]-glucopyranosyl-(1'''→2'')-*O*-α-l-arabinopyranoside-7-*O*-β-d-glucopyranoside (**4**). The structure elucidation was performed by MS, 1D and 2D NMR analyses.

## 1. Introduction

*Brugmansia suaveolens* (Humb. & Bonpl. ex. Willd.) Bercht. & C. Presl (Syn. *Datura suaveolens*), known also as angel’s trumpet, is a flowering shrub of the Solanaceae family, native to the coastal rainforest regions of Southeast Brazil. Extracts from its leaves have been studied for their anti-inflammatory and wound healing activities [1,2]. Up until now, alkaloids [3,4,5] and essential oils [6] have been reported as the main effective compounds. Moreover, in [7] the isolation of the flavonol glycosides kaempferol 3-*O*-α-l-arabinopyranoside and kaempferol 3-*O*-α-l-arabinopyranoside-7-*O*-β-d-glucopyranoside from the leaves of this species has been described. In this paper, the isolation and structure elucidation of four new kaempferol glycosides are reported for the first time.

## 2. Results and Discussion

**Figure 1:**
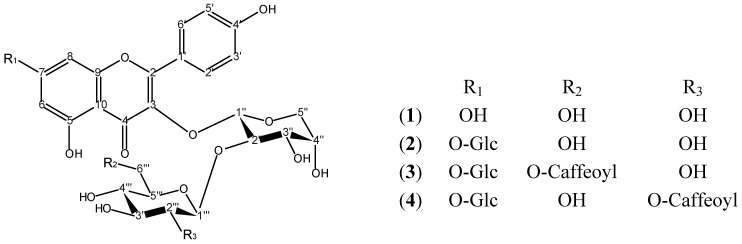
Chemical structures of compounds **1**–**4** isolated from the leaves of *Brugmansia suaveolens*.

Phytochemical investigation of the ethanolic extract of *B. suaveolens*, prepared from the leaves, yielded four new kaempferol glycosides, while neither of the compounds described by [7] were isolated in this work. Analysis of the spectroscopic data led to the identification of compounds **1**–**4** (Figure 1).

The molecular formula of compound **1** was determined as C_26_H_28_O_15_ on the basis of FT-ICR-MS *m/z* 603.132636 [M+Na]^+^ (calcd. *m/z* 603.13204 [M+Na]^+^) and ESI-MS *m/z* 603.1 [M+Na]^+^. The IR spectrum showed the characteristic absorption bands of hydroxyl (3248.2 cm^−1^), carbonyl (1652.8 cm^−1^), and phenyl groups (1574.4 cm^−1^). The ^1^H-NMR and ^13^C-NMR spectra (see Table 1) are similar to those reported by [7]. The aromatic region of the ^1^H-NMR spectrum of **1** showed four signals, namely, two broad singlets (δ 6.21 and δ 6.40) for the protons in ring A and two doublets (AA'BB' system) for ring B (δ 6.92 and δ 8.02, both with *J* = 8.6 Hz), which are typical for a kaempferol aglycone [7]. The middle region of the spectrum exhibited two anomeric signals due to the sugar units at δ 5.48 and δ 4.55. The coupling constant of the anomeric proton of the glucose (*J* = 7.8 Hz) was in accordance with a β-glycosidic linkage, while the coupling constant of the anomeric proton of the pentose (*J* = 3.3 Hz) indicated a α-glycosidic linkage [8]. Acid hydrolysis of **1** indicated d-glucose and l-arabinose, which were compared with an authentic sample by TLC analysis [9,10]. A further eleven protons were identified between δ 3.34 and δ 4.21 due to the sugar units [8]. The structural information concerning the attachment position of the aforementioned moieties was determined based on MS fragmentation and 2D-NMR analysis. The MS fragmentation of **1** showed no [M+H−pentose]^+^ fragment, indicating that the pentose is bonded directly to the aglycone, and that the glucose unit is linked as the second sugar moiety [11,12]. The HMBC experiment of **1** exhibited a correlation of the anomeric proton H-1'' of α-l-arabinopyranose (δ 5.48) with C-3 (δ_C3_ 135.7), indicating the linkage of this sugar with the aglycone moiety. The interglycosidic linkage is shown by the correlation of the H-1''' (δ 4.55) of β-d-glucopyranose with the C-2'' (δ_C2''_ 80.1) of α-l-arabinopyranose [13]. Based on the aforementioned spectroscopic data, the structure of **1** was assigned as a kaempferol 3-*O*-β-d-glucopyranosyl-(1'''→2'')-*O*-α-l-arabinopyranoside.

**Table 1:** ^1^H-NMR, ^13^C-NMR, and 2D-NMR spectral data for compounds **1** and **2**.

	1 ^a^					2 ^b^				
Position	δ_H_ ( *J* in Hz)		δ_C_	COSY	HMBC	δ_H_ ( *J* in Hz)		δ_C_	COSY	HMBC
**2**			158.5					155.9		
**3**			135.7					134.3		
**4**			179.7					177.7		
**5**			161.6					160.2		
**6**	6.21	Brs	99.9	H-8	C-7,8,10	6.44	Brs	99.3	H-8	C-5,7,8,10
**7**			166.0					162.9		
**8**	6.40	Brs	94.7	H-6	C-6,7,9,10	6.79	Brs	94.6	H-6	C-6,7,9,10
**9**			159.0					156.6		
**10**			105.8					105.6		
**1'**			122.6					120.3		
**2'**	8.02	d (8.6)	132.4	H-3'	C-2,4'	8.10	d (8.4)	131.1	H-3'	C-2,4'
**3'**	6.92	d (8.6)	116.5	H-2'	C-1′,4'	6.91	d (8.4)	115.4	H-2'	C-1',4'
**4'**			163.1					160.2		
**5'**	6.92	d (8.6)	116.5	H-6′	C-1',4'	6.91	d (8.4)	115.4	H-6'	C-1',4'
**6'**	8.02	d (8.6)	132.4	H-5′	C-2, 4'	8.10	d (8.4)	131.1	H-5′	C-2, 4'
**Ara-O-3**										
**1''**	5.48	d (3.3)	101.3	H-2''	C-3	5.61	d (3.1)	98.9	H-2''	C-3
**2''**	4.21	dd (5.4,3.4)	80.1	H-1'',3''		4.07	M	78.7	H-1'',3''	
**3′′**	3.96	dd (5.4,6.4)	71.3	H-2'',4''		3.86	M	68.7	H-2'',4''	
**4''**	3.86	M	66.6	H-3'',5''		3.70	M	64.1	H-3'',5''	
**5_a_''**	3.23	M	63.4	H-4′′		3.07	M	61.2	H-4''	
**5_b_′′**	3.73	M				3.51	brd (11.1)			
**Glc-Ara**										
**1'''**	4.55	d (7.8)	105.4	H-2'''	C-2''	4.37	d (7.5)	103.8	H-2'''	C-2''
**2'''**	3.25	brd (7.8)	75.2	H-1''',3'''		2.97	M	73.6	H-1''',3'''	
**3'''**	3.38	M	78.1	H-2''',4'''		3.17	M	76.7	H-2''',4'''	
**4'''**	3.34	M	71.4	H-3''',5'''		3.12	M	69.7	H-3''',5'''	
**5'''**	3.36	M	78.0	H-4''', 6'''		3.43	M	77.1	H-4''', 6'''	
**6_a_'''**	3.78–3.82	M	62.7	H-5'''		3.43	brd (11.5)	60.9	H-5′′′	
**6_b_'''**						3.59	M			
**Glc-O-7**										
**1''''**						5.07	d (7.1)	99.8	H-2''''	C-7
**2''''**						3.25	M	73.1	H-1'''',3''''	
**3''''**						3.30	M	76.4	H-2'''',4''''	
**4''''**						3.17	M	69.6	H-3'''',5'''',	
**5''''**						3.12	M	76.8	H-4'''',6''''	
**6_a_''''**						3.70	M	60.6	H-5''''	
**6_b_''''**						3.43	M			

^a^ MeOH-*d_4_*; ^b ^DMSO-*d_6_*; ^13^C-NMR measured in 100 MHz; ^1^H-NMR measured in 600 MHz.

Compound **2** was established as C_32_H_38_O_20_ on the basis of FT-ICR-MS *m/z* 765.184176 [M+Na]^+^ (calcd. *m/z* 765.18486 [M+Na]^+^) and ESI-MS *m/z* 765.0 [M+Na]^+^. The IR spectrum revealed signals of hydroxyl (3365.8 cm^−1^), carbonyl (1653.8 cm^−1^), and phenyl groups (1605.3 cm^−1^). The ^1^H-NMR and ^13^C-NMR (see Table 1) spectra are similar to those of **1**. In the middle region of the spectrum, one additional anomeric signal was identified, due to a new sugar unit at δ 5.07. The coupling constant of this anomeric proton (*J* = 7.1 Hz) was in accordance with a β-glycosidic linkage. The sugar units of compound **2** were determined as d-glucose and l-arabinose by TLC comparison with authentic samples after acid hydrolysis [9,10]. As in **1**, the HMBC experiment of **2** exhibited correlation between the anomeric proton H-1'' (δ 5.61) and C-3 (δ_C3_ 134.3), and between H-1'''' (δ 5.07) and C-7 (δ_C7_ 162.9), indicating, respectively, the linkage of α-l-arabinopyranose and of β-d-glucopyranose with the aglycone. Additionally, the interglycosidic linkage is shown by the correlation of the H-1''' (δ 4.37) of another β-d-glucopyranose with the C-2'' (δ_C2''_ 78.7) of α-l-arabinopyranose [13]. Therefore, compound **2** was identified as a kaempferol 3-*O*-β-d-glucopyranosyl-(1'''→2'')-*O*-α-l-arabino-pyranoside-7-*O*-β-d-glucopyranoside.

The molecular formula C_41_H_44_O_23_ of compound **3** was deduced based on FT-ICR-MS *m/z* 927.215977 [M+Na]^+^ (calcd. *m/z* 927.21656 [M+Na]^+^) and ESI-MS *m/z* 927.7 [M+Na]^+^. The absorption bands of the hydroxyl (3309.5 cm^−1^), carbonyl (1651.9 cm^−1^), and phenyl groups (1598.2 cm^−1^) were observed in IR analysis. The ^1^H-NMR and ^13^C-NMR spectra (see Table 2) were similar to those of **2**, including again three anomeric signals. Acid hydrolysis of **3** suggests d-glucose and l-arabinose as sugar moieties, which was confirmed by comparison with authentic samples by TLC analysis [9,10]. Additionally, 3 showed two further signals at δ 6.41 and δ 7.81 (both d, *J* = 15.8 Hz), which are indicative of olefinic protons of a *trans*-caffeoyl group [14,15]. The HMBC experiment exhibited correlation between the anomeric proton H-1'' of α-l-arabinopyranose (δ 6.41) and C-3 (δ_C3_ 137.0), and between the anomeric proton H-1'''''' (δ 5.78) and C-7 (δ_C7_ 163.6), showing direct linkages of these sugars moieties with aglycone. The correlation of the H-2'' (δ 5.07) of α-l-arabinopyranose with the C-1''' (δ_C1'''_ 106.7) of another β-d-glucopyranose indicates a linkage between these sugar moieties. The ^1^H-NMR spectrum of 3 exhibited the downfield shift of the signals corresponding to methylene protons H_2_-6''' to δ 4.90–5.03, indicating an acylation at this position [14]. The HMBC experiment confirmed the linkage of the caffeic acid to C-6''' of β-d-glucopyranose by a correlation between the H-6_a'''_ and H-6_b'''_ (δ 4.90–5.03) of this sugar unit and C=O (δ_C-1''''_ 167.4) of the caffeic acid moiety. Based on detailed analysis of the NMR spectra, the structure of compound 3 was determined as a kaempferol 3-*O*-β-d-[6'''-O-(E-caffeoyl)]-glucopyranosyl-(1'''→2'')-*O*-α-l-arabinopyranoside-7-*O*-β-d-glucopyranoside.

Compound **4** was determined as C_41_H_44_O_23_ based on FT-ICR-MS *m/z* 927.216170 [M+Na]^+^ (calcd. *m/z* 927.21656 [M+Na]^+^) and ESI-MS *m/z* 927.1 [M+Na]^+^. The IR spectrum showed bands of hydroxyl (3263.2 cm^−1^) and phenyl groups (1586.6 cm^−1^). The ^1^H-NMR and ^13^C-NMR spectra (see Table 2) are similar to those of **3**. Compound **4** showed again three anomeric signals and also two further signals at δ 6.25 and δ 7.47 (both d, *J* = 15.7 Hz), which were assigned to the olefinic protons of a *trans*-caffeoyl group [14,15]. Acid hydrolysis of **4**, followed by TLC comparison with authentic samples, indicated the presence of d-glucose and l-arabinose [9,10]. The HMBC experiment of **4** exhibited correlation between the anomeric proton H-1'' of α-l-arabinopyranose (δ 5.58) and C-3 (δ_C3_ 134.5), and between the anomeric proton H-1'''''' of a β-d-glucopyranose unit (δ 5.06) and C-7 (δ_C7_ 162.9), showing the linkages of these sugar units to the kaempferol moiety. The interglycosidic linkage is shown by the correlation of H-1''' (δ 4.68) of another β-d-glucopyranose with C-2'' (δ_C2''_ 78.7) of α-l-arabinopyranose [13]. The ^1^H-NMR spectrum of **4** exhibited also a downfield shift of the signal corresponding to H-2''' at δ 4.60, indicating an acylation at the C-2''' position. The HMBC experiment confirmed the assignment of the caffeic acid moiety to C-2''' of β-d-glucopyranose by a correlation between the H-2''' (δ 4.60, t, *J* = 8.7 Hz) of this sugar unit and the C=O (δ_C-1''''_ 165.6) of the caffeic acid moiety.

**Table 2:** ^1^H-NMR, ^13^C-NMR, and 2D-NMR spectral data for compounds 3 and 4.

	3 ^c^					4 ^b^					
Position	δ_H_( *J* in Hz)		δ_C_	COSY	HMBC	δ_H_( *J* in Hz)		δ_C_	COSY	HMBC	
**2**			157.2					154.4			
**3**			137.0					134.5			
**4**			178.8					177.6			
**5**			161.7					160.5			
**6**	6.72	brs	100.0	H-8	C-5,7,8,10	6.43	Brs	98.7	H-8	C-7	
**7**			163.6					162.9			
**8**	6.92	brs	94.6	H-6	C-6,7,9,10	6.76	Brs	94.0	H-6	C-7,10	
**9**			156.6					155.9			
**10**			106.7					106.0			
**1'**			121.8					121.2			
**2'**	8.44	d (8.6)	131.8	H-3'	C-2,4'	8.06	d (8.6)	131.0	H-3'	C-2	
**3'**	7.26	d (8.6)	116.2	H-2'	C-1',4'	6.81	d (8.6)	115.4	H-2'	C-1',4'	
**4'**			162.0					160.5			
**5'**	7.26	d (8.6)	116.2	H-6'	C-1',4'	6.81	d (8.6)	115.4	H-6'	C-1',4'	
**6'**	8.44	d (8.6)	131.8	H-5'	C-2,4'	8.06	d (8.6)	131.0	H-5'	C-2	
**Ara-O-3**											
**1''**	6.41	d (5.0)	100.4	H-2''	C-3	5.58	d (3.4)	98.7	H-2''	C-3	
**2''**	5.07	m	80.7	H-1'',3''		4.09	M	78.7	H-1'',3''		
**3''**	4.67	m	70.9	H-2'',4''		3.85	M	68.6	H-2'',4''		
**4''**	4.47	m	66.0	H-3'',5''		3.50	M	63.7	H-3'',5''		
**5_a_''**	4.38	m	62.1	H-4''		3.00	brd (11.6)	60.6	H-4''		
**5_b_''**	4.56	brd (11.9)				3.50	M				
**Glc-Ara**											
**1'''**	5.28	d (7.5)	106.7	H-2'''	C-2''	4.68	d (7.8)	101.5	H-2'''	C-2''	
**2'''**	4.12	m	75.1	H-1''',3'''		4.60	M	73.1	H-1''',3'''	C-1''''	
**3'''**	4.05	m	78.1	H-2''',4'''		3.48	M	73.6	H-2''',4'''		
**4'''**	4.15	m	70.9	H-3''',5'''		3.25	M	69.5	H-3''',5'''		
**5'''**	4.05	m	75.4	H-4''',6'''		3.30	M	76.4	H-4''',6'''		
**6_a_'''**	4.90–5.03	m	63.9	H-5'''	C-1''''	3.50–3.70	M	60.3	H-5'''		
**6_b_'''**											
**Caffeoyl**											
**1''''**			167.4					165.6			
**2''''**	6.41	d (15.8)	114.6	H-3''''	C1'''''	6.25	d (15.7)	113.9	H-3''''	C1'''''	
**3''''**	7.81	d (15.8)	145.6	H-2''''	C-1′′′′, 2'''''	7.47	d (15.7)		145.0		H-2''''		C-1'''', 2'''''		
**1'''''**			126.6					125.4		
**2'''''**	7.40	brs	115.6		C-3''''	7.07	Brs	114.9		C-3''''
**3'''''**			145.6					145.7		
**4'''''**			147.2					148.7		
**5'''''**	7.12	d (8.0)	116.3	H-6'''''		6.90	d (8.6)	115.8	H-6'''''	
**6'''''**	6.95	d (8.0)	121.8	H-5'''''		6.96	brd (8.0)	121.2	H-5'''''	
**Glc-O-7**										
**1''''''**	5.78	d (7.2)	101.3	H-2''''''	C-7	5.06	d (7.4)	99.8	H-2''''''	C-7
**2''''''**	4.30	m	74.6	H-1'''''',3''''''		3.20	M	72.5	H-1'''''',3''''''	
**3''''''**	4.40	m	78.9	H-2'''''',4''''''		3.28	M	76.8	H-2'''''',4''''''	
**4''''''**	4.30	m	71.0	H-3'''''',5''''''		3.25	M	69.8	H-3'''''',5''''''	
**5''''''**	4.30	m	78.1	H-4'''''',6''''''		3.40	M	77.1	H-4'''''',6''''''	
**6_a_''''''**	3.71	m	63.4	H-5''''''		3.50-3.70	M	60.3	H-5''''''	
**6_b_''''''**	4.50	m								

^b^ DMSO-*d_6_*; ^c^ Pyridine-*d_5_*; ^13^C-NMR measured in 100 MHz; ^1^H-NMR measured in 600 MHz.

Therefore, the structure of **4** was assigned as a kaempferol 3-*O*-β-d-[2'''-O-(E-caffeoyl)]-glucopyranosyl-(1'''→2'')-*O*-α-l-arabinopyranoside-7-*O*-β-d-glucopyranoside.

## 3. Experimental Section

### 3.1. General

NMR spectra 1D (^1^H, ^13^C, and DEPT-135) and 2D (^1^H-^1^H COSY, HSQC, HMBC) were recorded with MeOH-*d_4_*, DMSO-*d_6_* or pyridine-*d_5_* on Bruker AMX-600 (600 MHz and 150 MHz) and Bruker AMX-400 (400 MHz and 100 MHz) spectrometers (Bruker BioSpin GmbH, Rheinstetten, Germany). LC-ESI-MS was carried out on an electrospray Finnigan MAT P4000 HPLC-DAD system connected to a Finningan LCQ^TM^ Duo ion Trap mass spectrometer (Thermo Electron GmbH, Karlsruhe, Germany). Analytical HPLC was performed with a L-7100 Pump (Merck-HITACHI-LaChrom); UV-Vis detector L7420 (Merck-HITACHI-LaChrom); HPLC D-7000 HSM software with a D7000 data interface. HR-MS (FT-ICR) was performed on a Bruker APEXII (electrospray ionization). The UV spectra were measured in a Hewlett-Packard HP 1090 HPLC-DAD system in MeOH. The IR spectra were obtained on a Perkin Elmer Spectrum One (ATR Technology, Shelton, CT, USA). Sephadex LH-20 (6 × 50 cm) (particle size 25–100 mm, Sigma Chemical Co., Munich, Germany); Thin layer chromatography (TLC) (Silica Gel 60 F254 (0.25 mm) Merck (Darmstadt, Germany); RP-TLC aluminum sheets 20 × 20 cm, RP-18 F254 Merck); Flash chromatography (LaFlash, VWR International, Darmstadt, Germany), RP-18 (25–40 μm) column (5 × 20 cm), with gradient MeOH–H_2_O (10:90 ➔ 100:0, *v/v*); Analytical HPLC, LiChrospher RP-18 column (5 × 100 mm; 5 µm), mobile phase (A) MeOH–ACN–FA (95:5:0.1, *v/v/v*) and (B) gradient of MeOH–ACN–FA (95:5:0.1➔85:15:0.1, *v/v/v*).

### 3.2. Plant Material

Leaves of *Brugmansia suaveolens* were collected in Santa Maria, Rio Grande do Sul, Brazil in January 2008. The plant was identified by botanist Gilberto Zanetti. A voucher specimen was deposited in the herbarium of the Department of Biology at the Federal University of Santa Maria, Brazil, under reference number SMDB12520.

### 3.3. Extraction and Isolation

The air-dried and powdered leaves (1.087 g) of *B. suaveolens* were exhaustively extracted with EtOH (7 L, 30 h) in a Soxhlet apparatus. The EtOH extract was concentrated under vacuum at 40 °C and lyophilized to yield 359.94 g, which was treated with MeOH at −20 °C giving a soluble fraction of 344.82 g after solvent removal. Of this extract, 6.18 g was subjected to column chromatography with Sephadex LH-20 and MeOH as mobile phase at a flow rate of 1 mL/min. A total of 300 fractions of 10 mL each were collected and controlled by TLC using silica gel with toluene–MeOH–DEA (8:1:1, *v/v/v*) and RP-18 with MeOH–H_2_O (1:1, *v/v*); Anisaldehyde-H_2_SO_4_ was used for detection. Those fractions with a similar profile were combined, yielding 11 fractions (A➔K). Fraction G (80 mg) was chromatographed by RP-18 CC using MeOH–H_2_O (1:1, *v/v*) to give five combined sub-fractions (G_1.1_-G_1.5_). A sub-fraction G_1.2_ was fractioned again by RP-18 CC with MeOH–H_2_O (1:2, *v/v*) to provide three sub-fractions (G_1.2.1_-G_1.2.3_). Sub-fraction G_1.2.1_ was further purified by HPLC using a gradient of MeOH–ACN–FA (95:5:0.1➔85:15:0.1, *v/v/v*), yielding compound **2** (10.3 mg). Fraction I (41.1 mg) was chromatographed by RP-18 gel CC using MeOH–H_2_O (1:1, *v/v*) to give three combined sub-fractions (I_1.1_-I_1.3_). Sub-fraction I_1.2.2_ was further purified by HPLC using a gradient of MeOH–ACN–FA (95:5:0.1➔85:15:0.1, *v/v/v*) to afford compound **3** (4.5 mg). Fraction H (100 mg) was separated by flash chromatography RP-18 with gradient MeOH–H_2_O (10:90 ➔ 100:0, *v/v*) to give four combined sub-fractions (H_1.1_-H_1.4_). Sub-fraction H_1.4_ (47.8 mg) was further purified by RP-18 CC using MeOH–H_2_O (1:1, *v/v*) yielding 3.2 mg of compound **1**. Sub-fraction H_1.2_ (35.2 mg) was applied to HPLC using a gradient of MeOH–ACN–FA (95:5:0.1➔85:15:0.1, *v/v/v*), affording 11.1 mg of compound 3 and 3.5 mg of compound **4**.

### 3.4. Acid Hydrolysis

An amount of 2 mg per compound **1**–**4** was dissolved in EtOH–HCl 10% (10 mL) and refluxed at 80 °C for 2 h. The mixture was diluted in water (10 mL) and extracted with EtOAc (3 × 3 mL). The aglycone and sugar moieties were identified by TLC analysis and compared with authentic samples, d(+)-glucose, l(+)-arabinose, and kaempferol used as standards (Sigma-Aldrich, Munich, Germany). Silica gel (Merck), mobile phase for aglycone CHCl_3_–EtOAc–MeOH (14:3:3, *v/v/v*) detected with AlCl_3_, and mobile phase for sugars EtOH 96%–NH_4_OH 25%–H_2_O (20:1:4, *v/v/v*) detected with aniline phthalate [9,10].

*Kaempferol 3-O-β-d-glucopyranosyl-(1'''→2'')-O-α-l-arabinopyranoside* (**1**). Yellow amorphous powder. ESI-MS: positive ions m/z (rel. int.): 603.1 [M+Na]^+^ (54); 581.0 [M+H]^+^ (52); 419.0 [M+H−glucose]^+^ (43); 401.1 [M+H−glucose−H_2_O]^+^ (13); 287.2 [aglycone+H]^+^ (100). FT-ICR-MS (ESI): [Measured: 603.132636]^+^ (calculated mass for C_26_H_28_O_15_Na^+^: 603.13204). UV λ max (nm) MeOH: 265, 346. ^1^H-NMR, ^13^C-NMR, COSY, and HMBC: Table 1.

*Kaempferol 3-O-β-d-glucopyranosyl-(1′′′→2′′)-O-α-l-arabinopyranoside-7-O-β-d-glucopyranoside* (**2**). Yellow amorphous powder. ESI-MS: positive ions m/z (rel. int.): 765.0 [M+Na]^+^ (10); 742.8 [M+H]^+^ (22); 580.9 [M+H−glucose]^+^ (29); 448.8 [M+H−arabinose−glucose]^+^ (100); 418.9 [M+H−glucose−glucose]^+^ (100); 400.9 [M+H−glucose−glucose−H_2_O]^+^ (7); 287.1 [aglycone+H]^+^ (82). FT-ICR-MS (ESI): [Measured: 765.184176]^+^ (calculated mass for C_32_H_38_O_20_Na^+^: 765.18486). UV λ max (nm) MeOH: 265, 345. ^1^H-NMR, ^13^C-NMR, COSY, and HMBC: Table 1.

*Kaempferol 3-O-β-d-[6′′′-O-(E-caffeoyl)]-glucopyranosyl-(1′′′→2′′)-O-α-l-arabinopyranoside-7-O-β-d-glucopyranoside* (**3**). Yellow amorphous powder. ESI-MS: positive ions m/z (rel. int.): 927.7 [M+Na]^+^ (13); 904.8 [M+H]^+^ (74); 742.9 [M+H−caffeic acid]^+^ (21); 580.8 [M+H−caffeic acid−glucose]^+^ (24); 448.9 [M+H−caffeic acid−arabinose−glucose]^+^ (100); 418.8 [M+H−caffeic acid−glucose−glucose]^+^ (7); 324.8 [caffeic acid+glucose−H]^+^ (8); 287.1 [aglycone+H]^+^ (37); 162.9 [caffeic acid−OH]^+^ (8). FT-ICR-MS (ESI): [Measured: 927.215977]^+^ (calculated mass for C_41_H_44_O_23_Na^+^: 927.21656). UV λ max (nm) MeOH: 265, 328. ^1^H-NMR, ^13^C-NMR, COSY, and HMBC: Table 2.

*Kaempferol 3-O-β-d-[2′′′-O-(E-caffeoyl)]-glucopyranosyl-(1′′′→2′′)-O-α-l-arabinopyranoside-7-O-β-d-glucopyranoside* (**4**). Yellow amorphous powder. ESI-MS: positive ions m/z (rel. int.): 927.1 [M+Na]^+^ (42); 904.9 [M+H]^+^ (26); 742.9 [M+H−caffeic acid]^+^ (10); 581.0 [M+H−caffeic acid−glucose]^+^ (14); 448.9 [M+H−caffeic acid−arabinose−glucose]^+^ (49); 419.1 [M+H−caffeic acid−glucose−glucose]^+^ (6); 324.9 [caffeic acid+glucose−H]^+^ (25); 287.2 [aglycone+H]^+^ (100); 162.9 [caffeic acid−OH]^+^ (24). UV λ max (nm) MeOH: 265, 330. FT-ICR-MS (ESI): [Measured: 927.216170]^+^ (calculated mass for C_41_H_44_O_23_Na^+^: 927.21656). ^1^H-NMR, ^13^C-NMR, COSY, and HMBC: Table 2.

## 4. Conclusions

The four kaempferol glycosides isolated from the leaves of *B. suaveolens* are described for the first time in Nature. The similarities of the chemical structures suggest a common biosynthetic pathway with the constituents reported by [7] for the same plant species. Additional studies considering their biosynthesis should be performed to clarify this issue.

## Supplementary Materials

Supplementary materials can be accessed at: http://www.mdpi.com/1420-3049/19/5/6727/s1.
