# Supplementary material for: Four New Flavonol Glycosides from the Leaves of Brugmansia suaveolens
Source: Molecules. 2014 May 22;19(5):6727–36. doi: 10.3390/molecules19056727 (PMC6271595; doi:10.3390/molecules19056727)

Supporting Information

Figure S1. Extraction and isolation of compounds from ethanolic extract of the leaves of *Brugmansia suaveolens*.

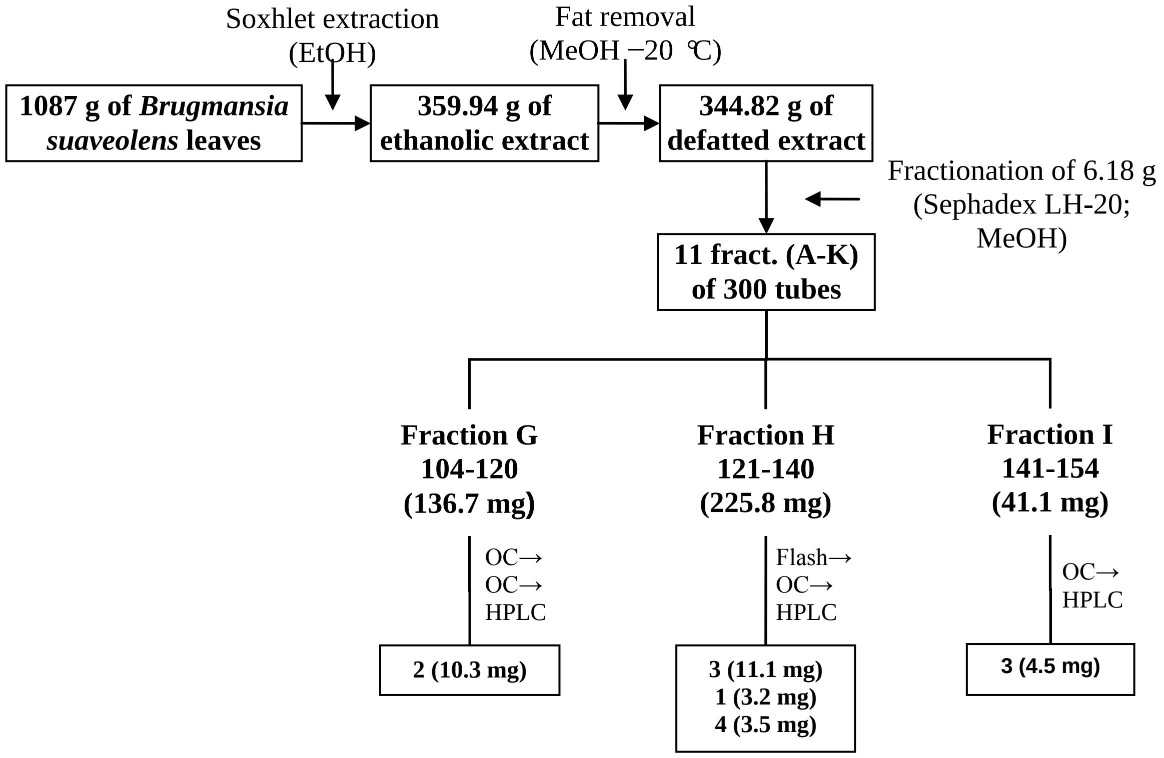

Figure S2. ESI-MS (positive mode) of compound **1**.

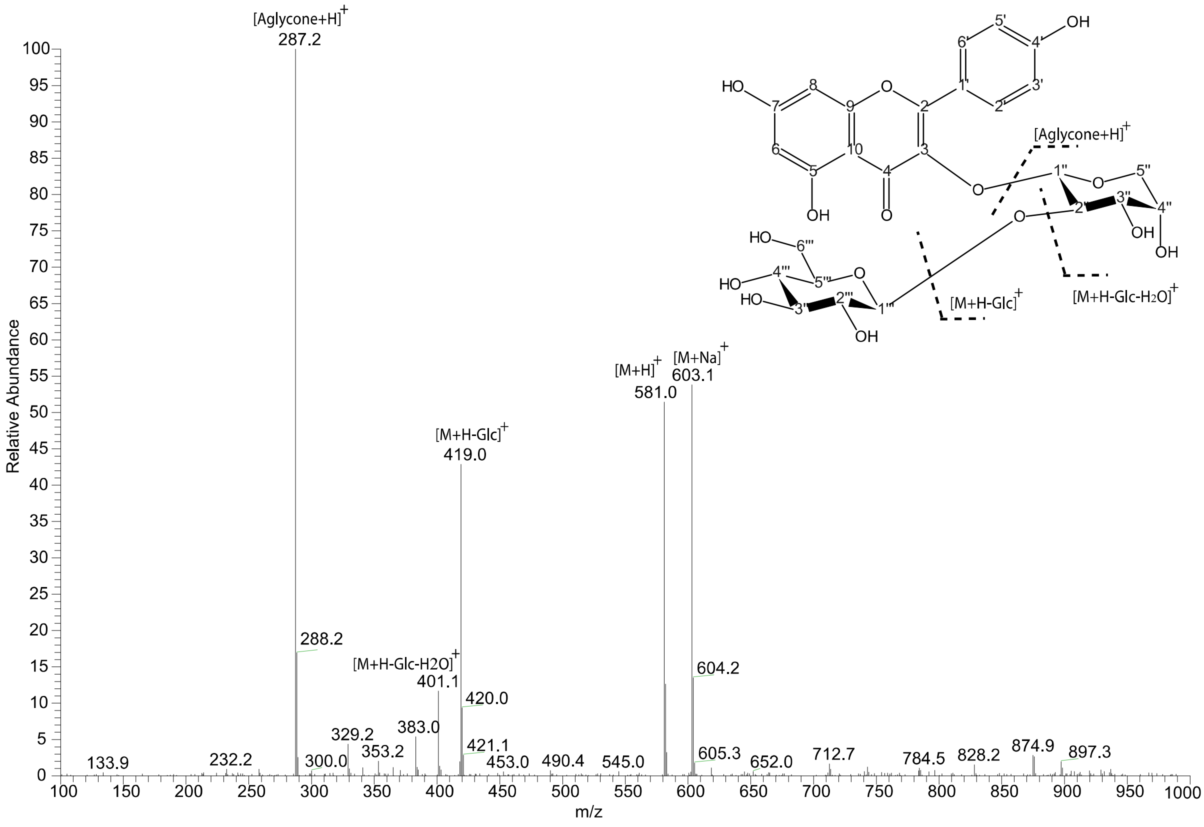

Figure S3. ^{1}H-NMR of **1** (600 MHz, MeOH-d_4).

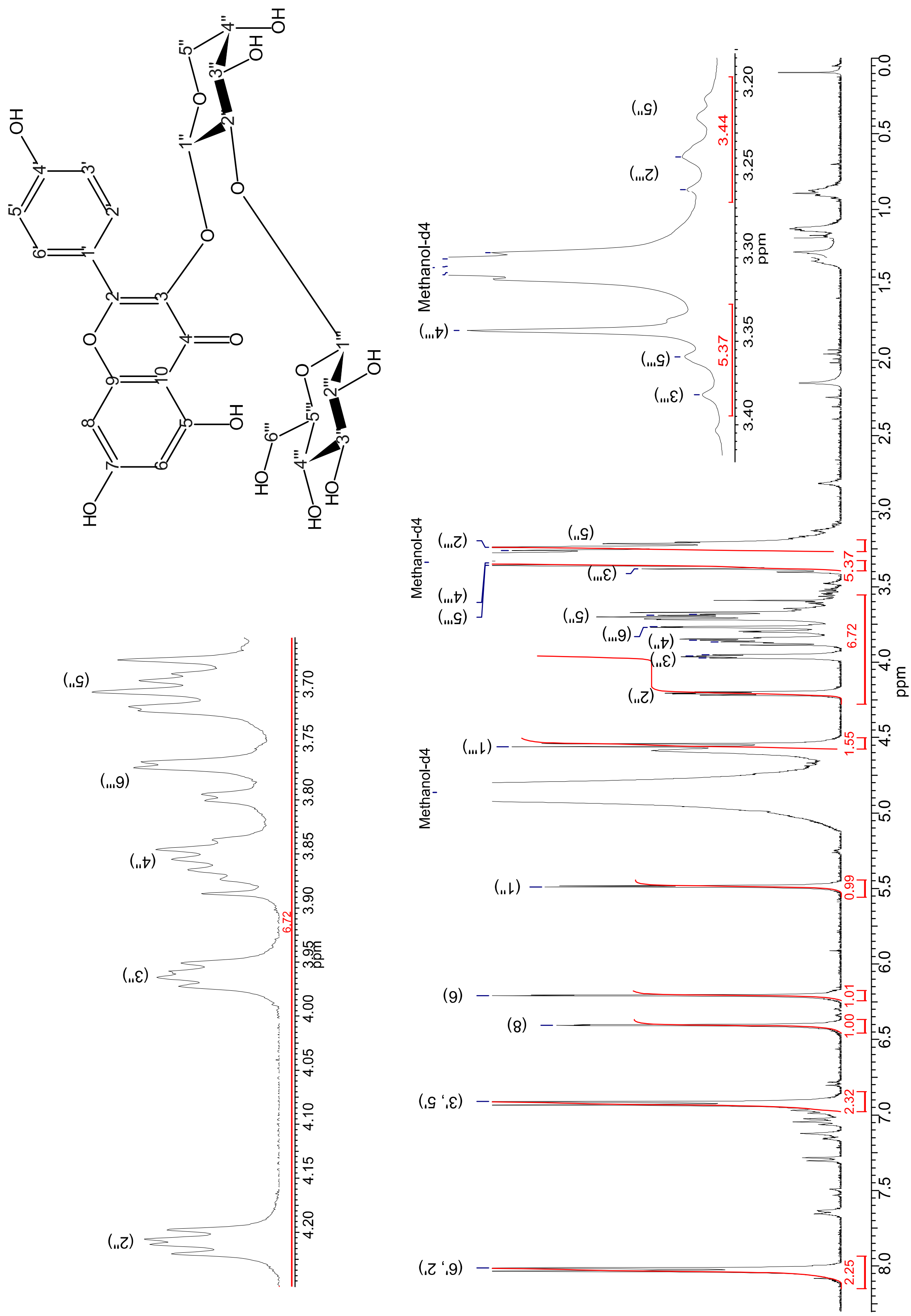

Figure S4. ^{13}C-NMR of **1** (100 MHz, MeOH-d_4).

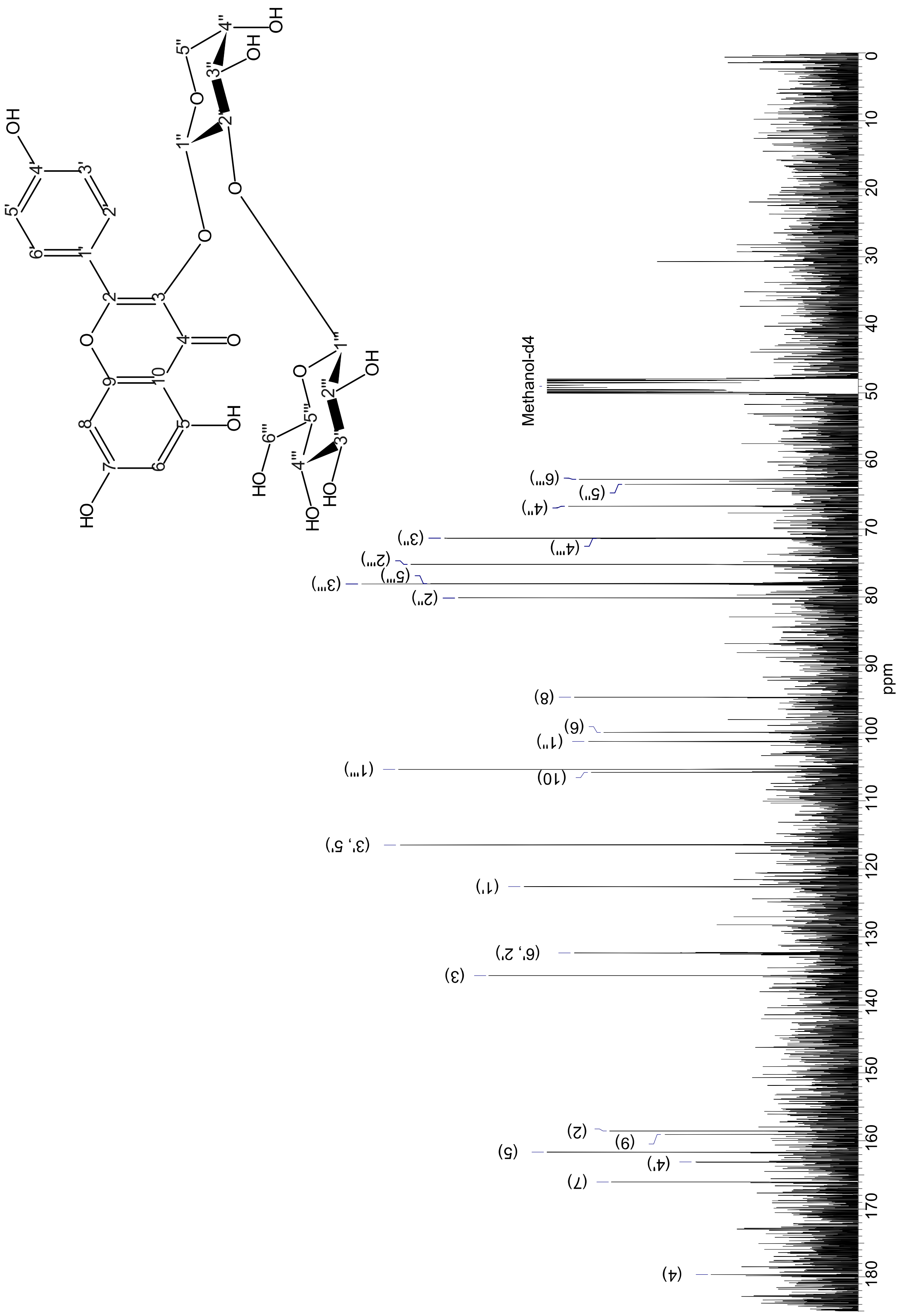

Figure S5. DEPT-135 of **1** (100 MHz, MeOH-d_4).

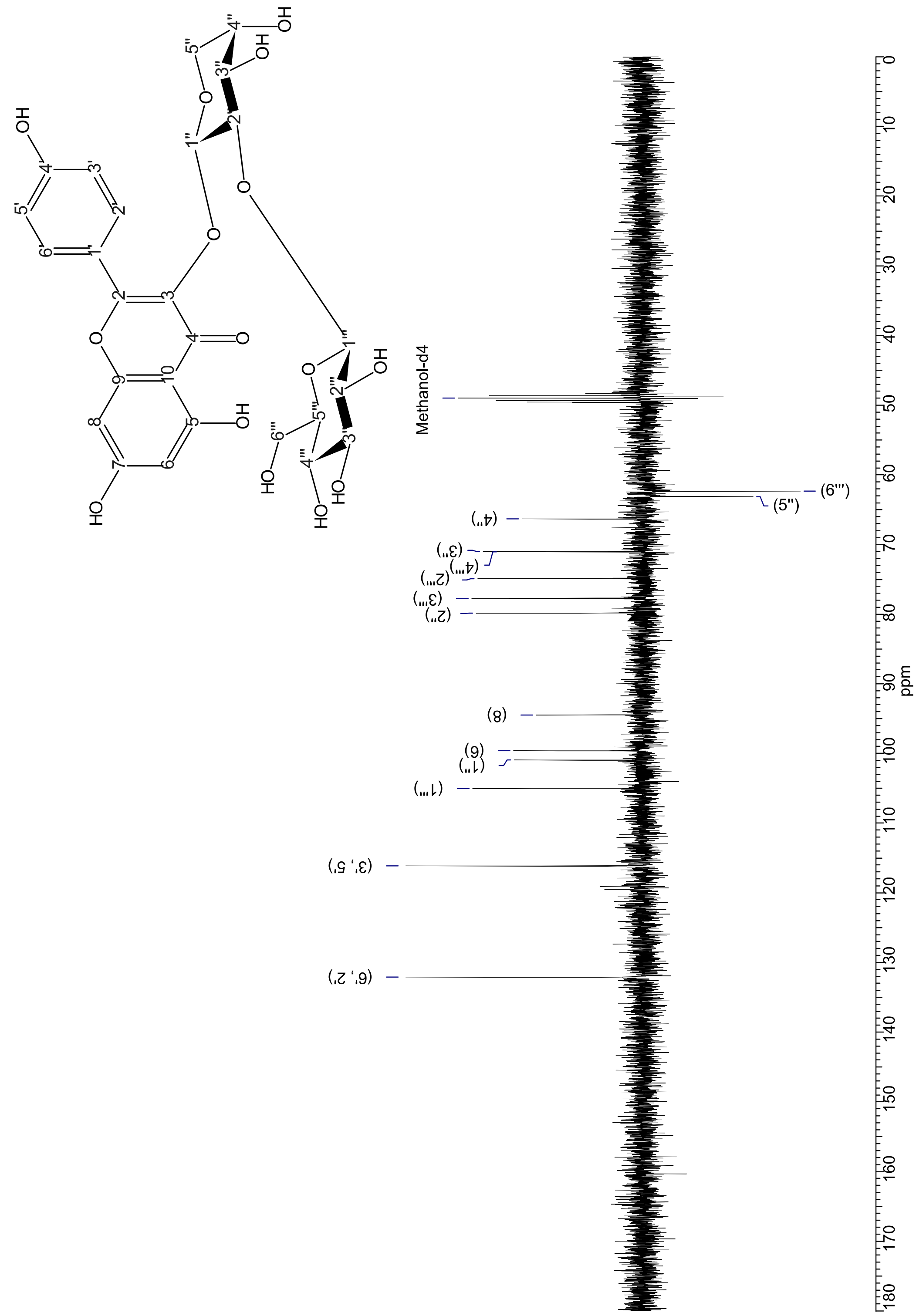

Figure S6. H-H-COSY of **1** (600 MHz, MeOH-d_4).

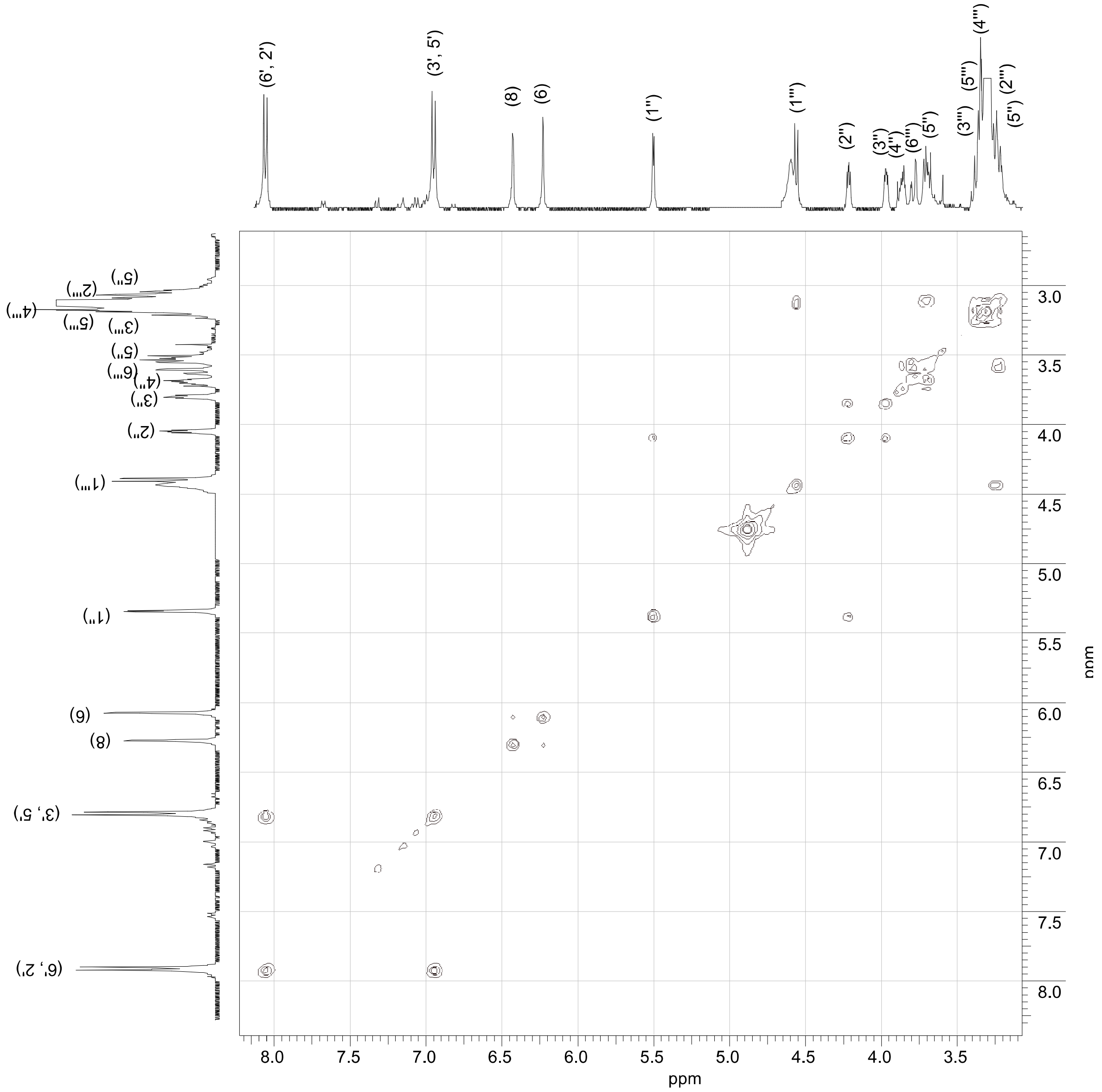

Figure S7. HMBC of **1** (600 MHz, MeOH-d_4).

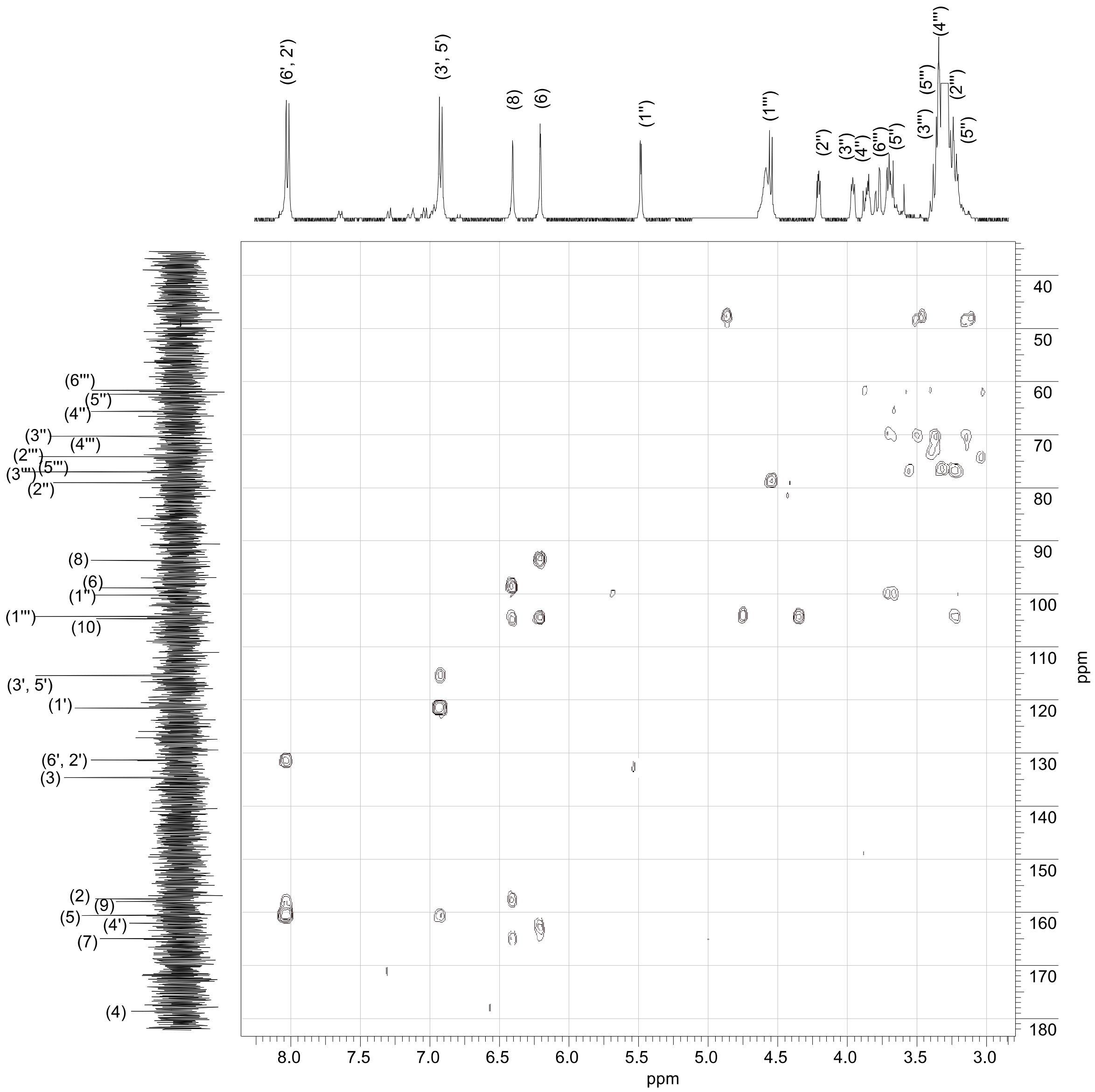

Figure S8. ESI-MS (positive mode) of compound **2**.

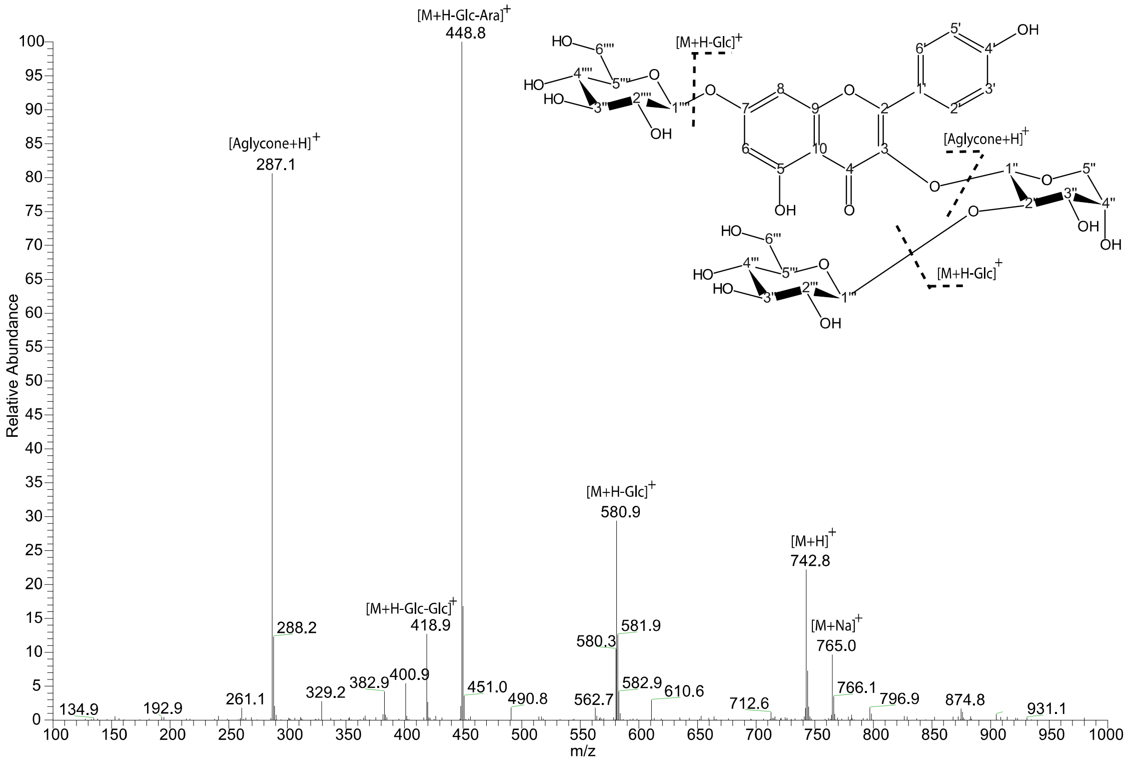

Figure S9. ^{1}H-NMR of **2** (600 MHz, DMSO-d_6).

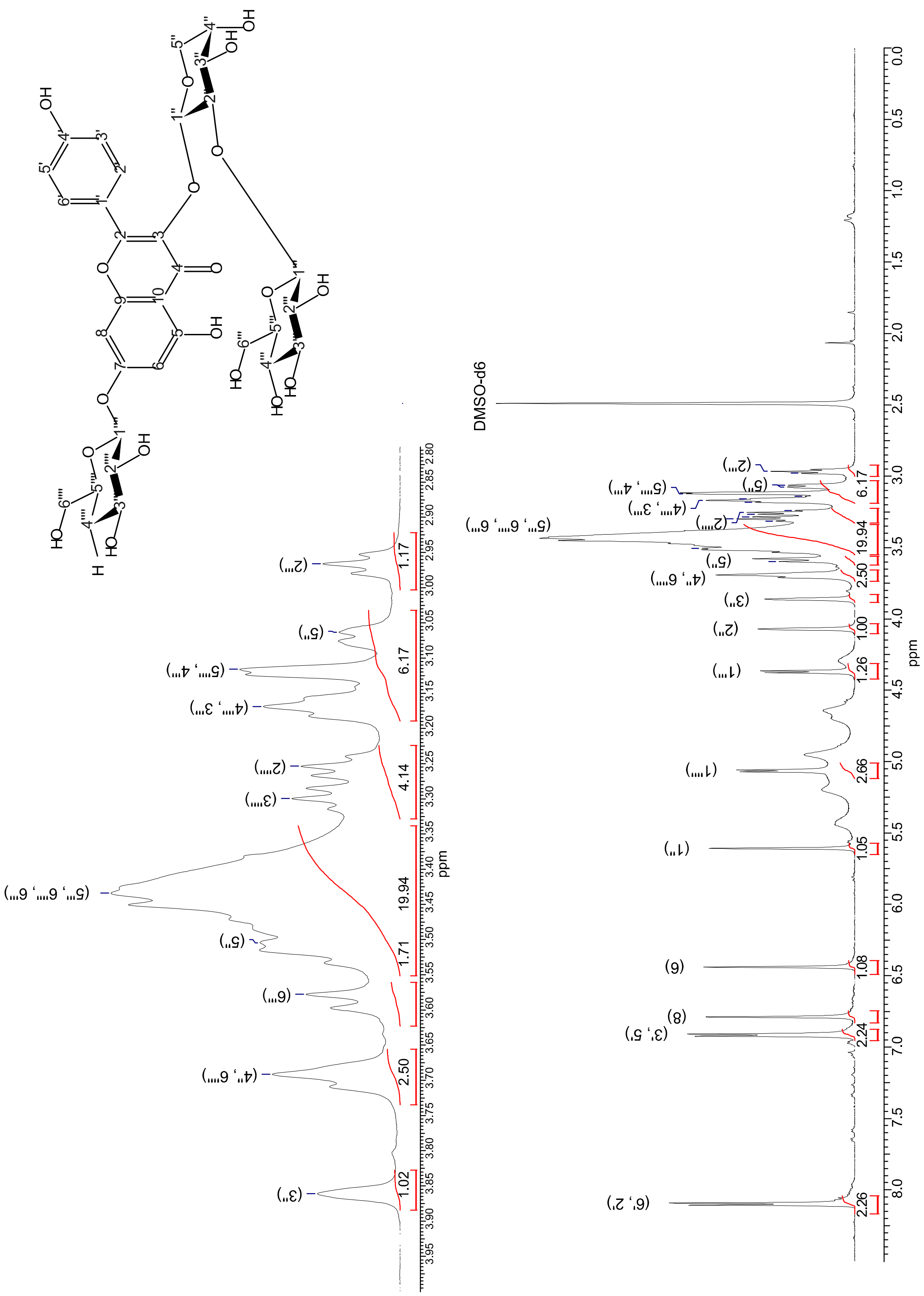

Figure S10. ^{13}C-NMR of **2** (100 MHz, DMSO-d_6).

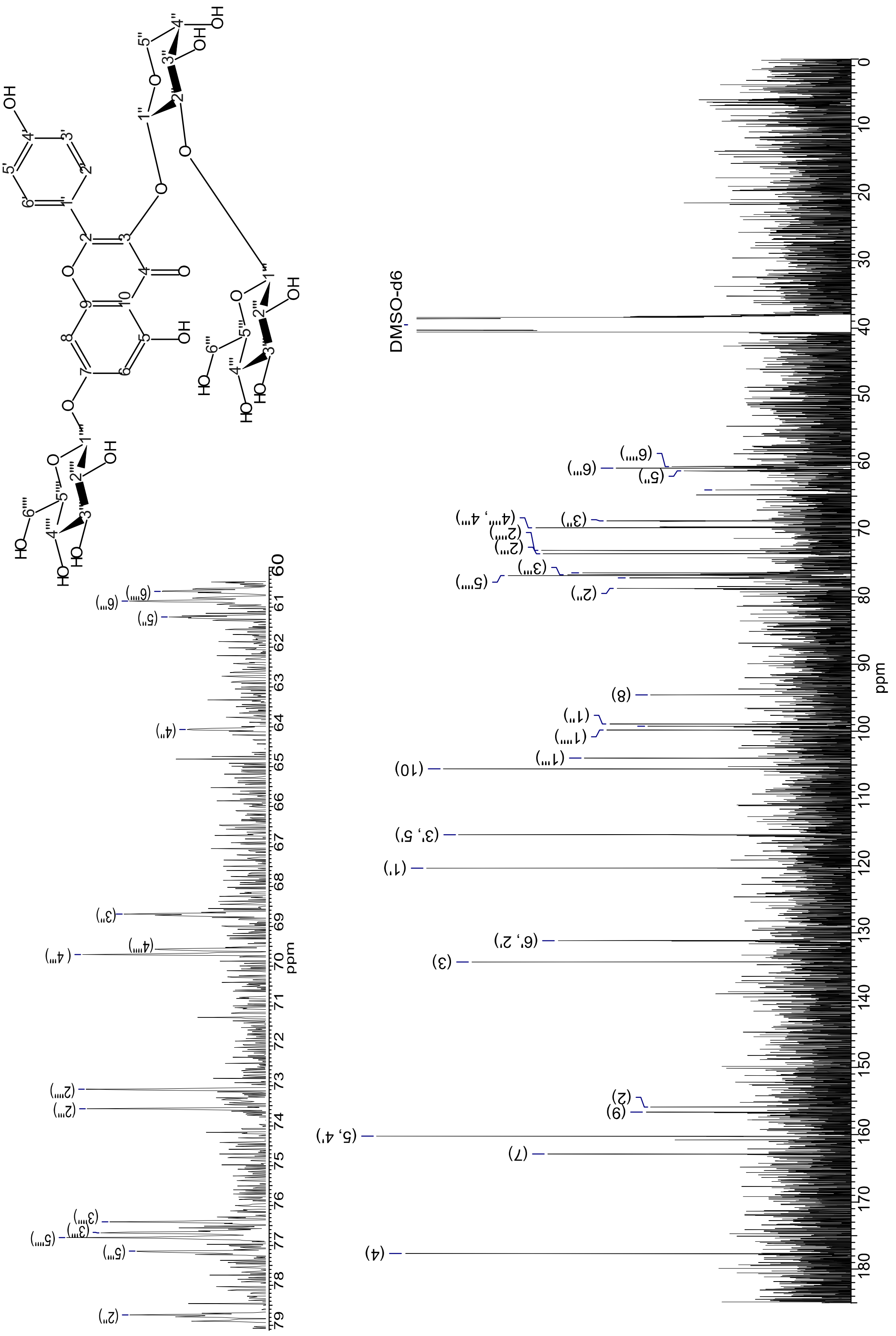

Figure S11. DEPT-135 of **2** (100 MHz, DMSO-d_6).

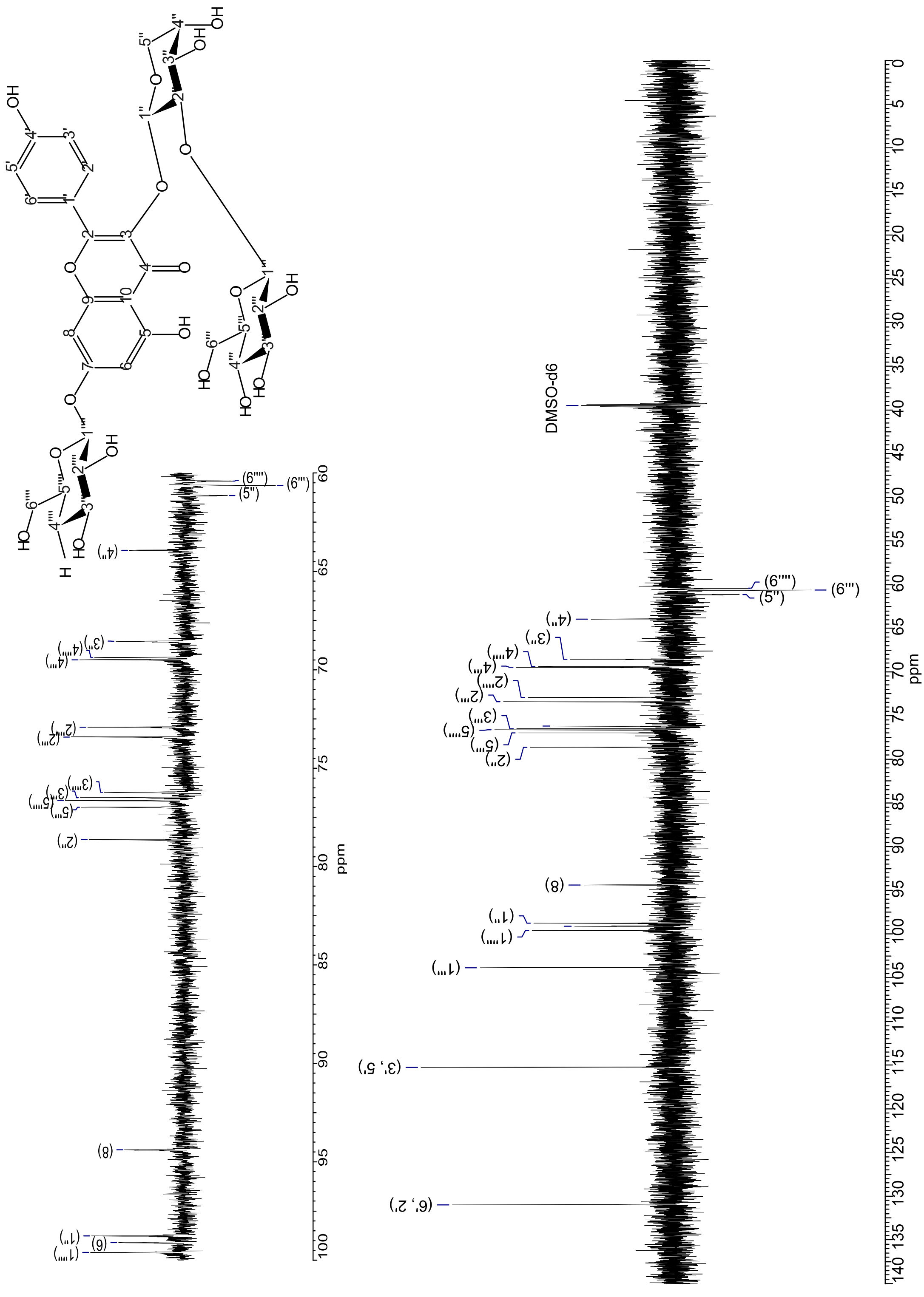

Figure S12. H-H-COSY of **2** (600 MHz, DMSO-d_6).

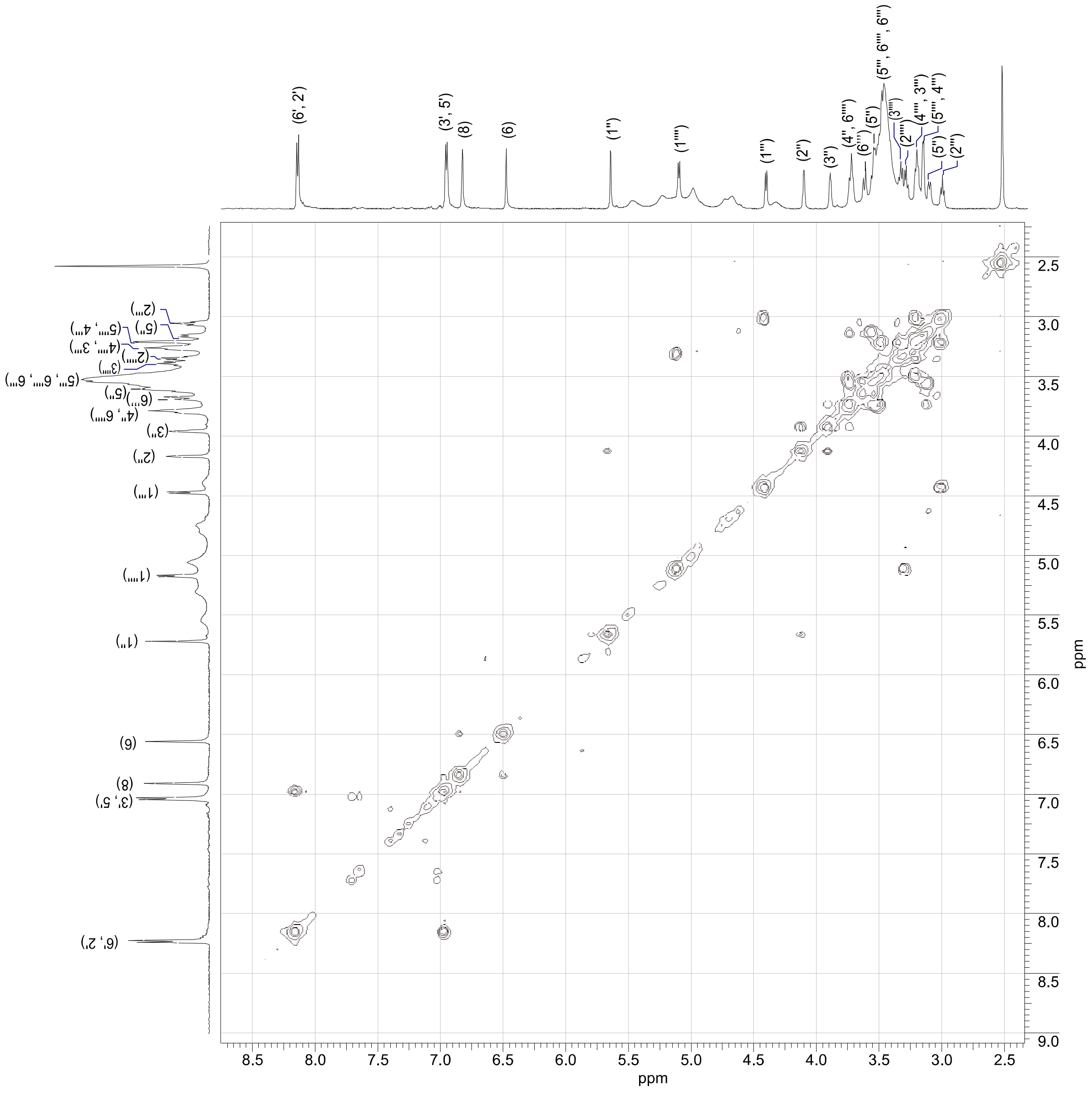

Figure S13. HSQC of **2** (600 MHz, DMSO-d_6).

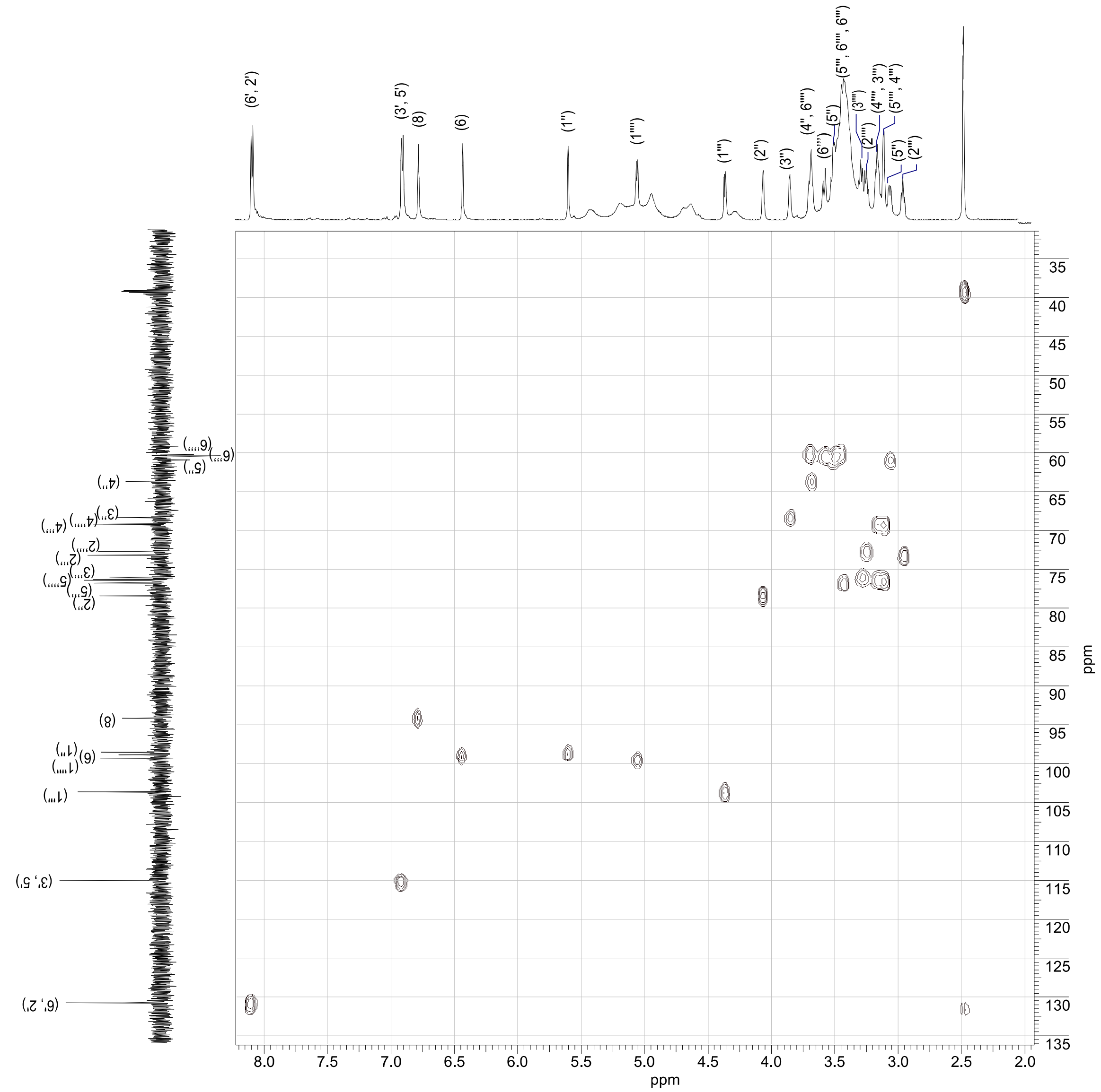

Figure S14. HMBC of **2** (600 MHz, DMSO-d_6).

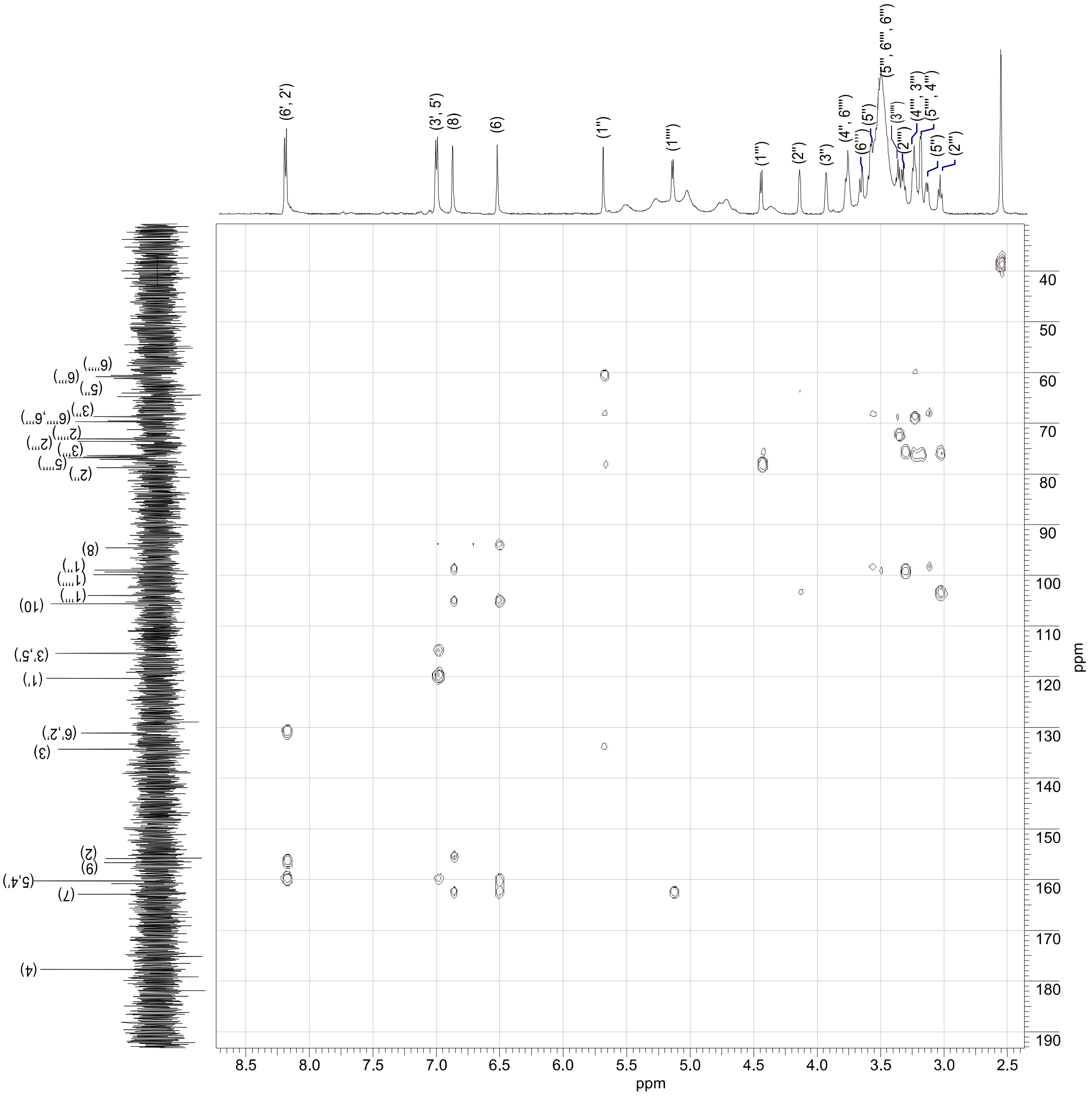

Figure S15. ESI-MS (positive mode) of compound **3**.

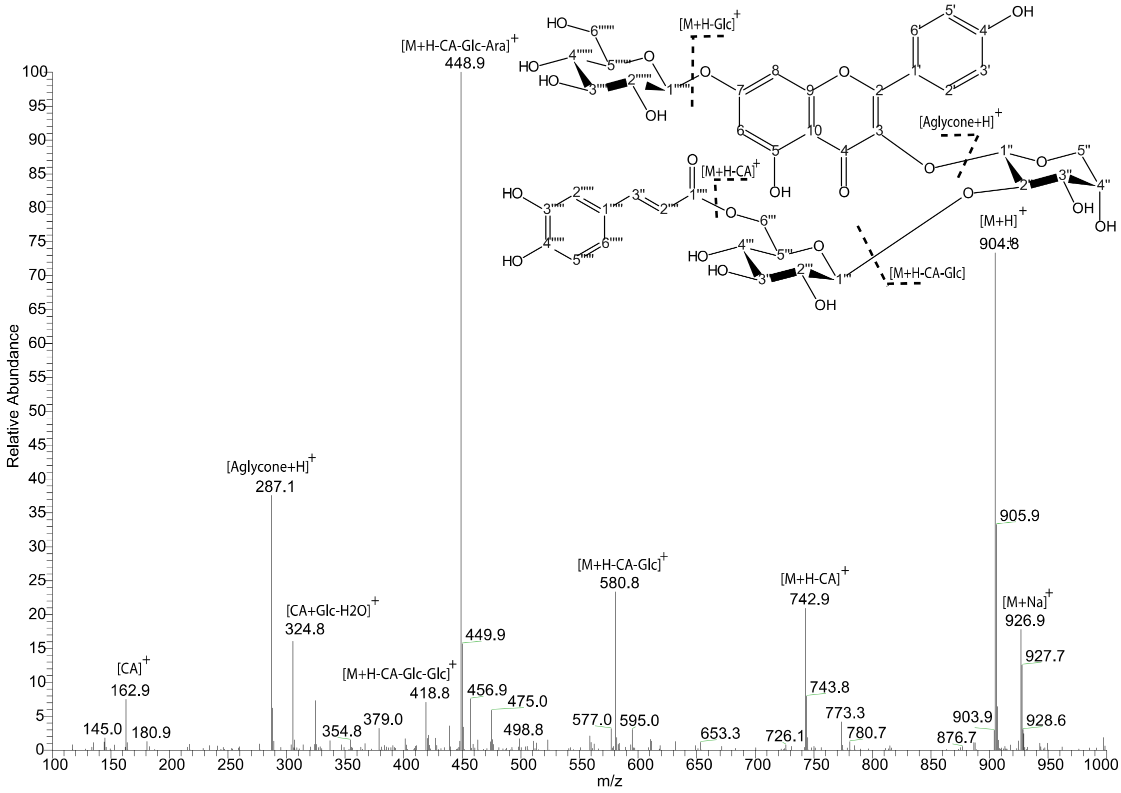

Figure S16. ^{1}H-NMR of **3** (600 MHz, Pyridine-d_5).

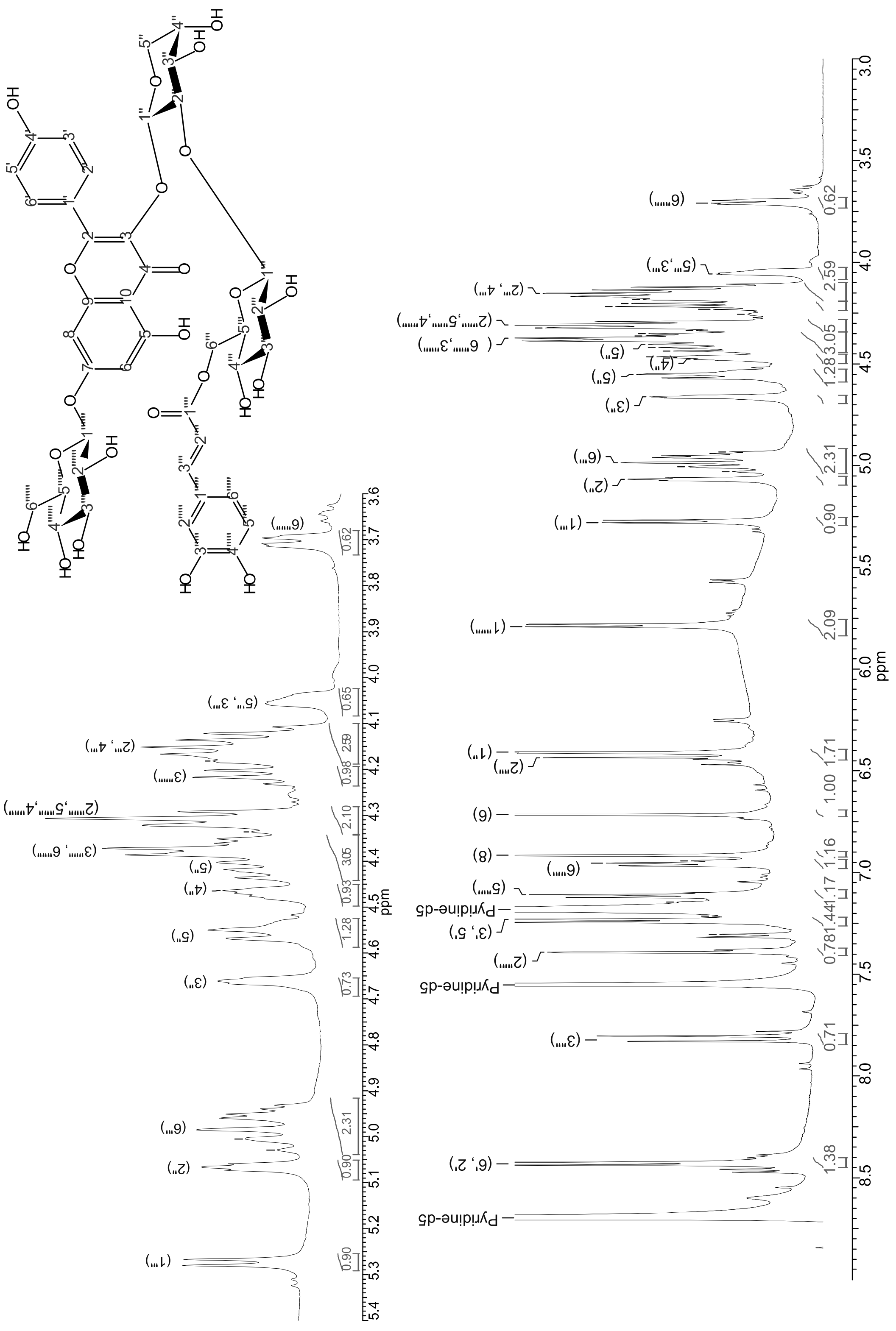

Figure S17. ^{13}C-NMR of **3** (100 MHz, Pyridine-d_5).

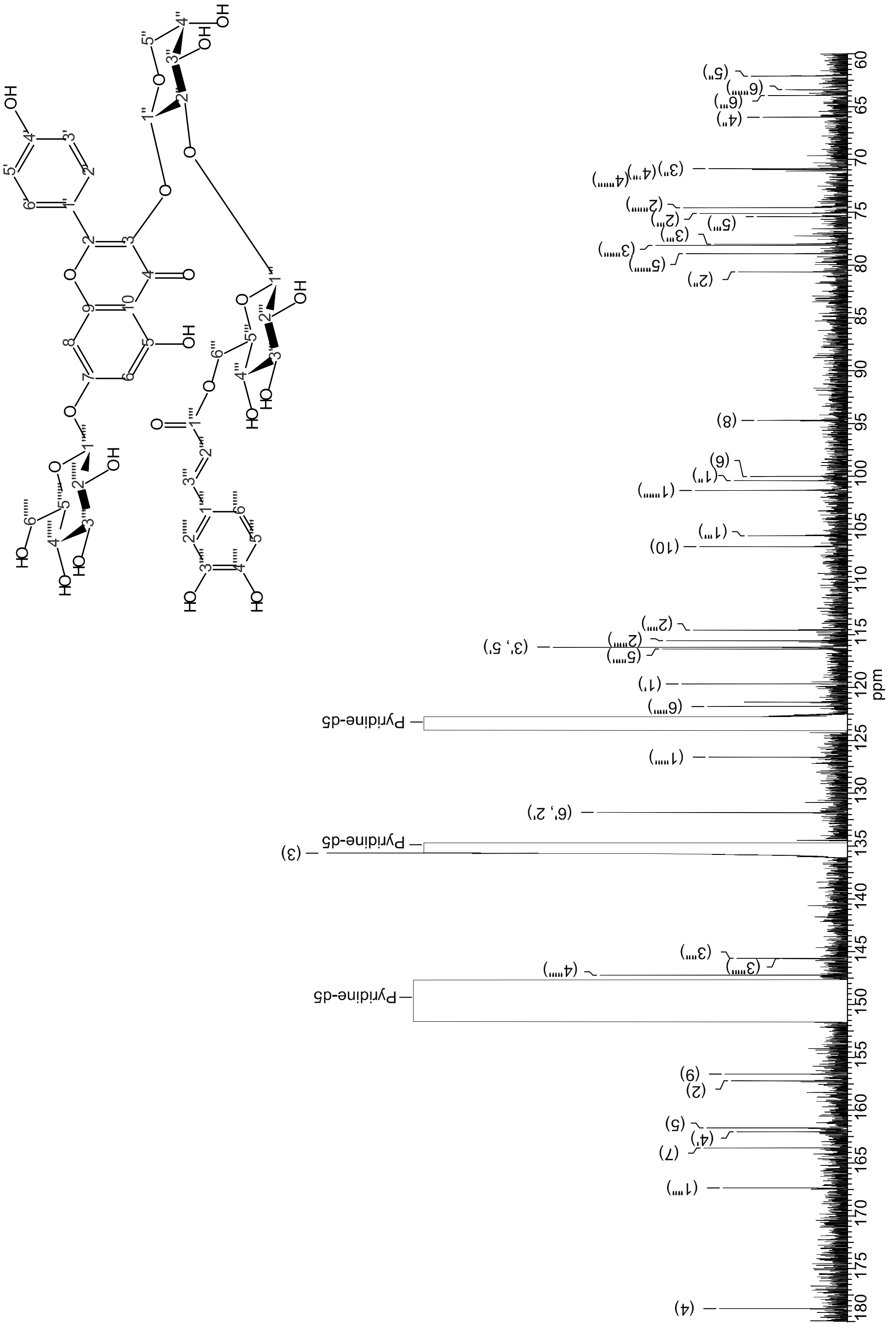

Figure S18. H-H-COSY of **3** (600 MHz, Pyridine-d_5).

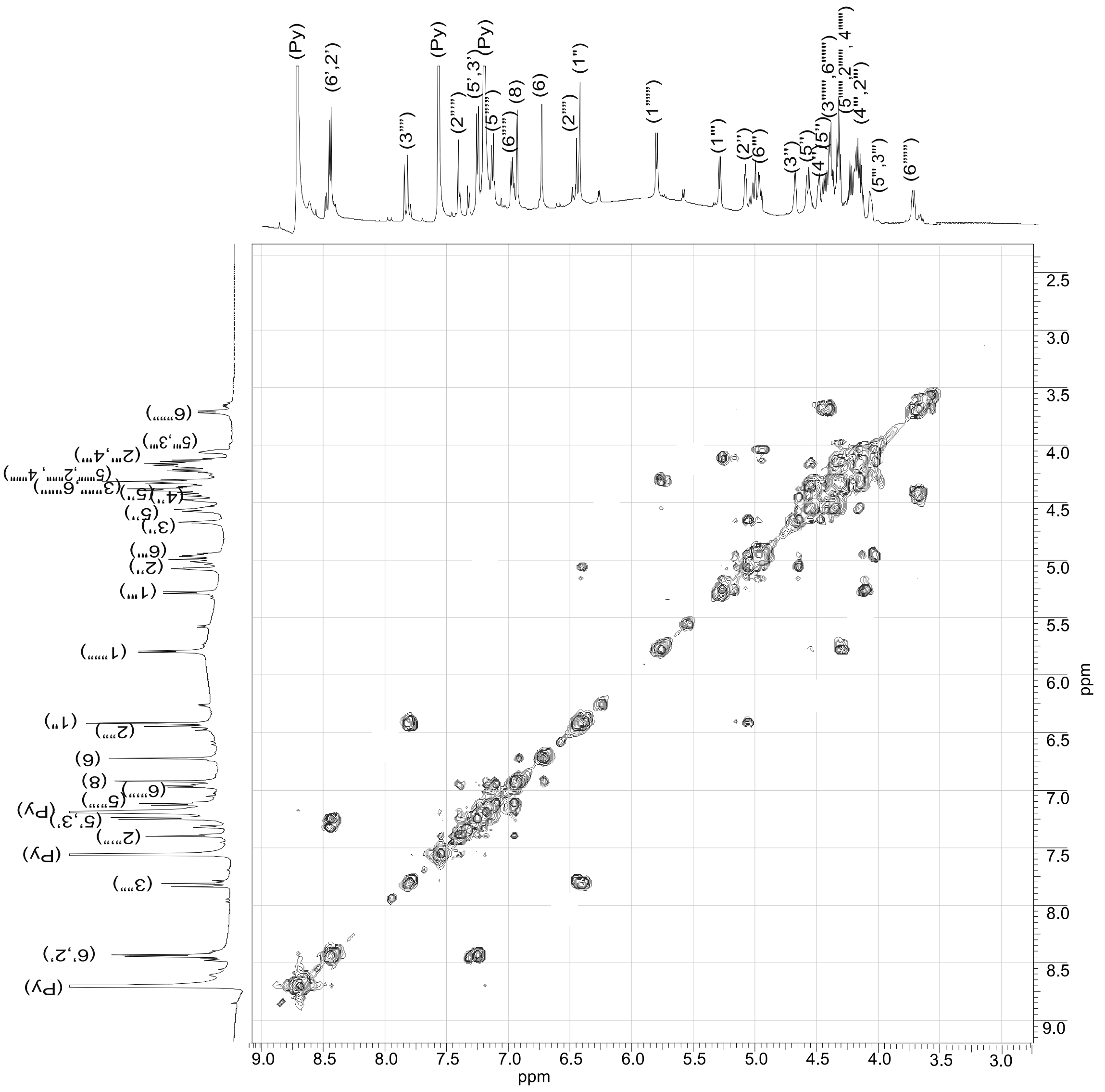

Figure S19. HSQC of **3** (600 MHz, Pyridine-d_5).

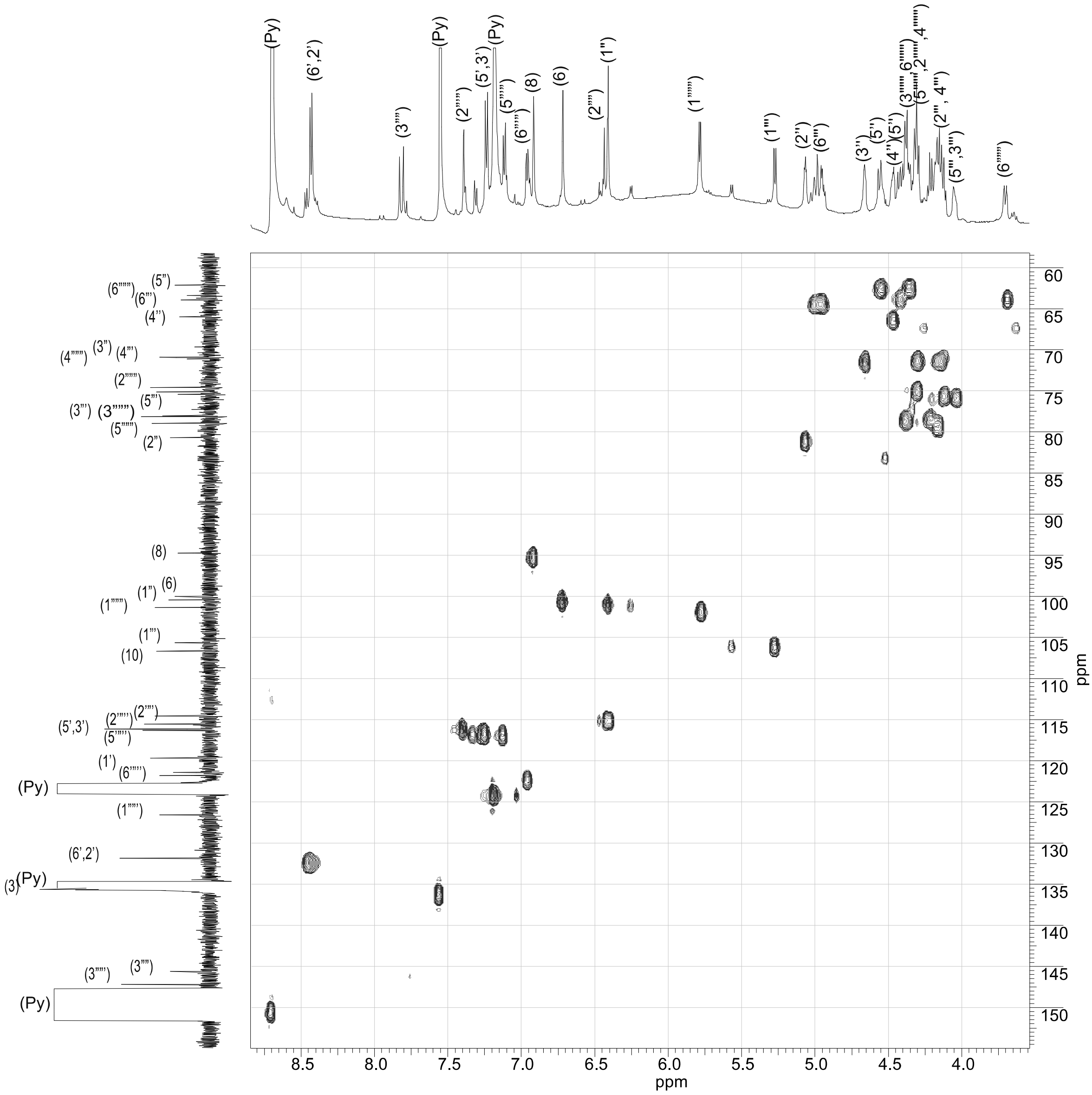

Figure S20. HMBC of **3** (600 MHz, Pyridine-d_5).

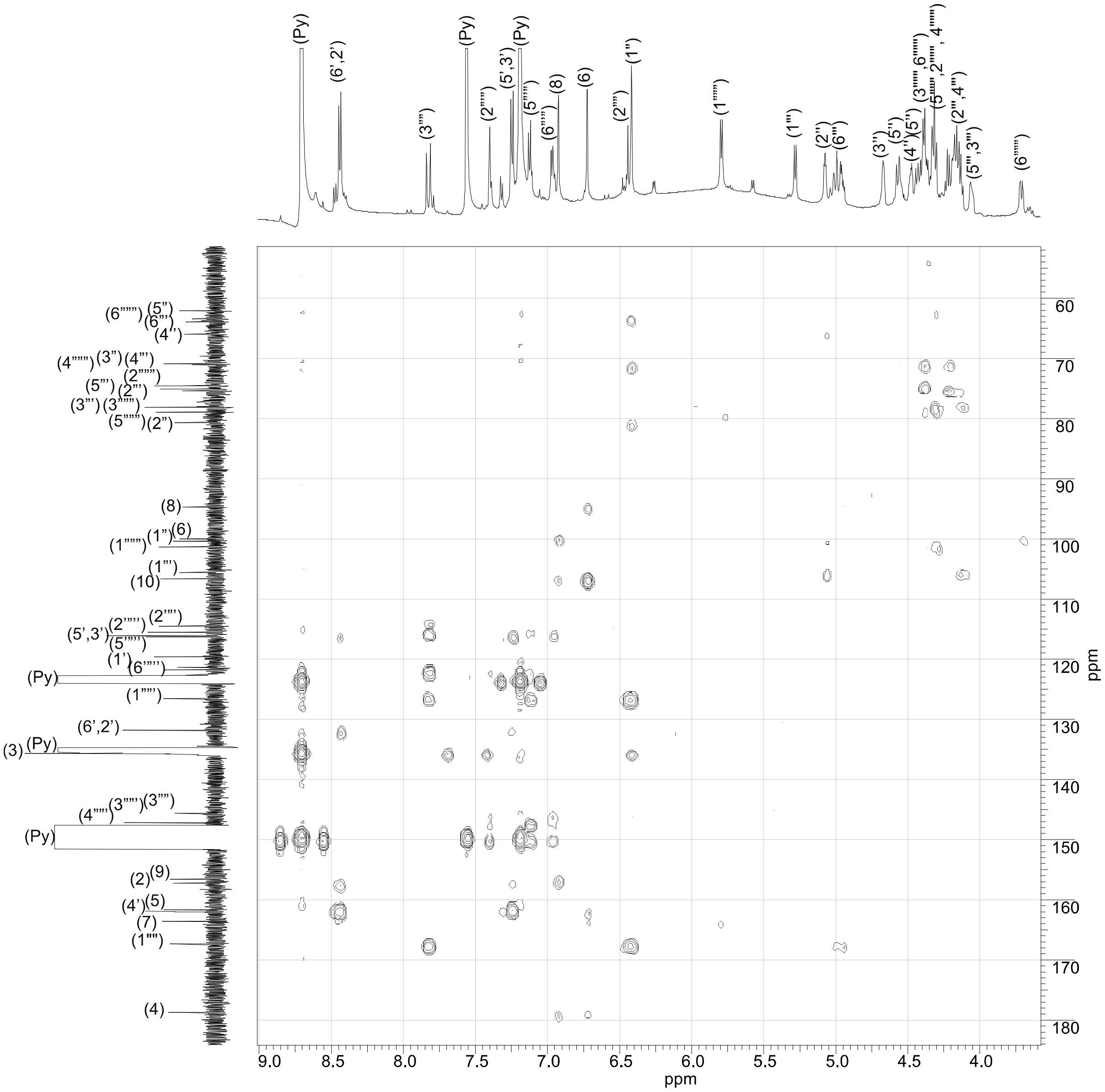

Figure S21. ESI-MS (positiv mode) of compound **4**.

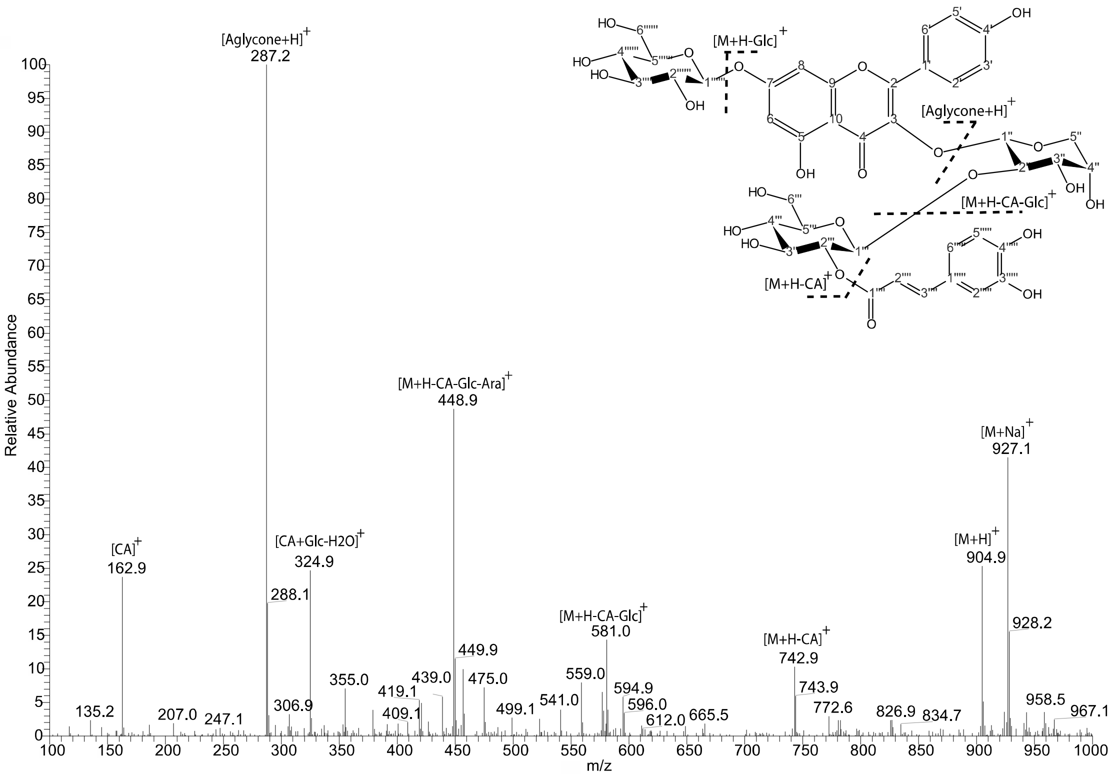

Figure S22. ^{1}H-NMR of **4** (600 MHz, DMSO-d_6).

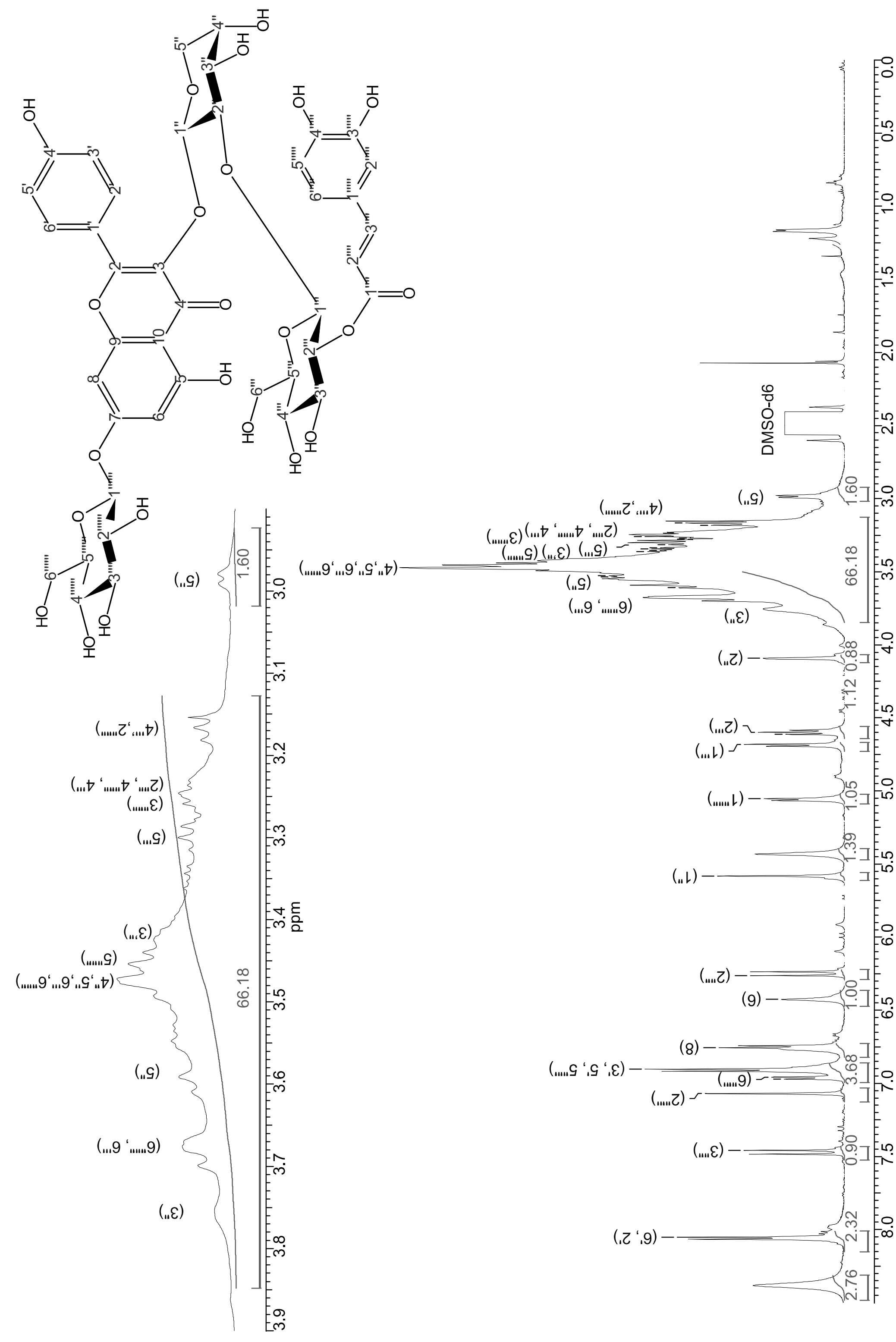

Figure S23. ^{13}C-NMR of **4** (100 MHz, DMSO-d_6).

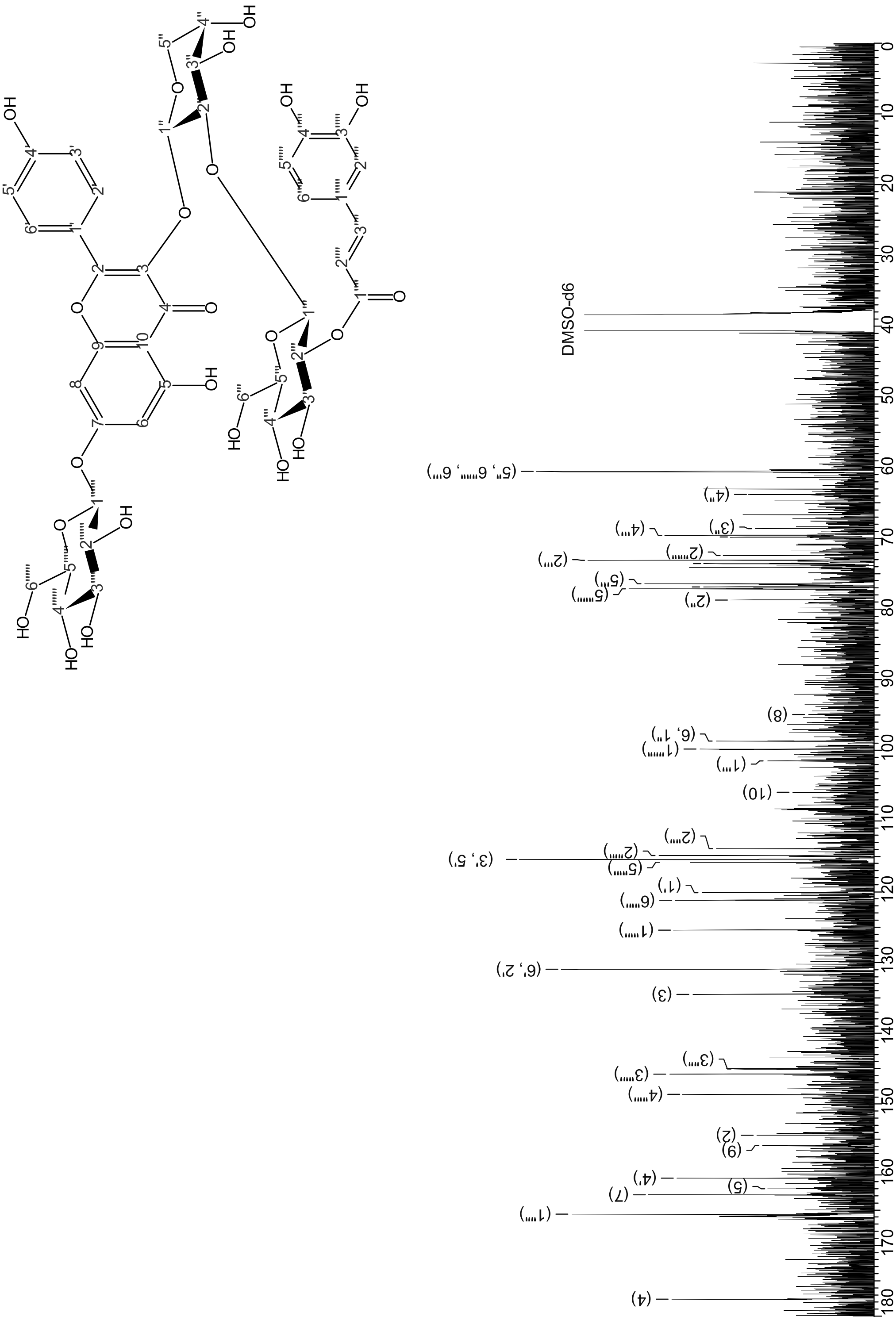

Figure S24. DEPT-135 of **4** (100 MHz, DMSO-d_6).

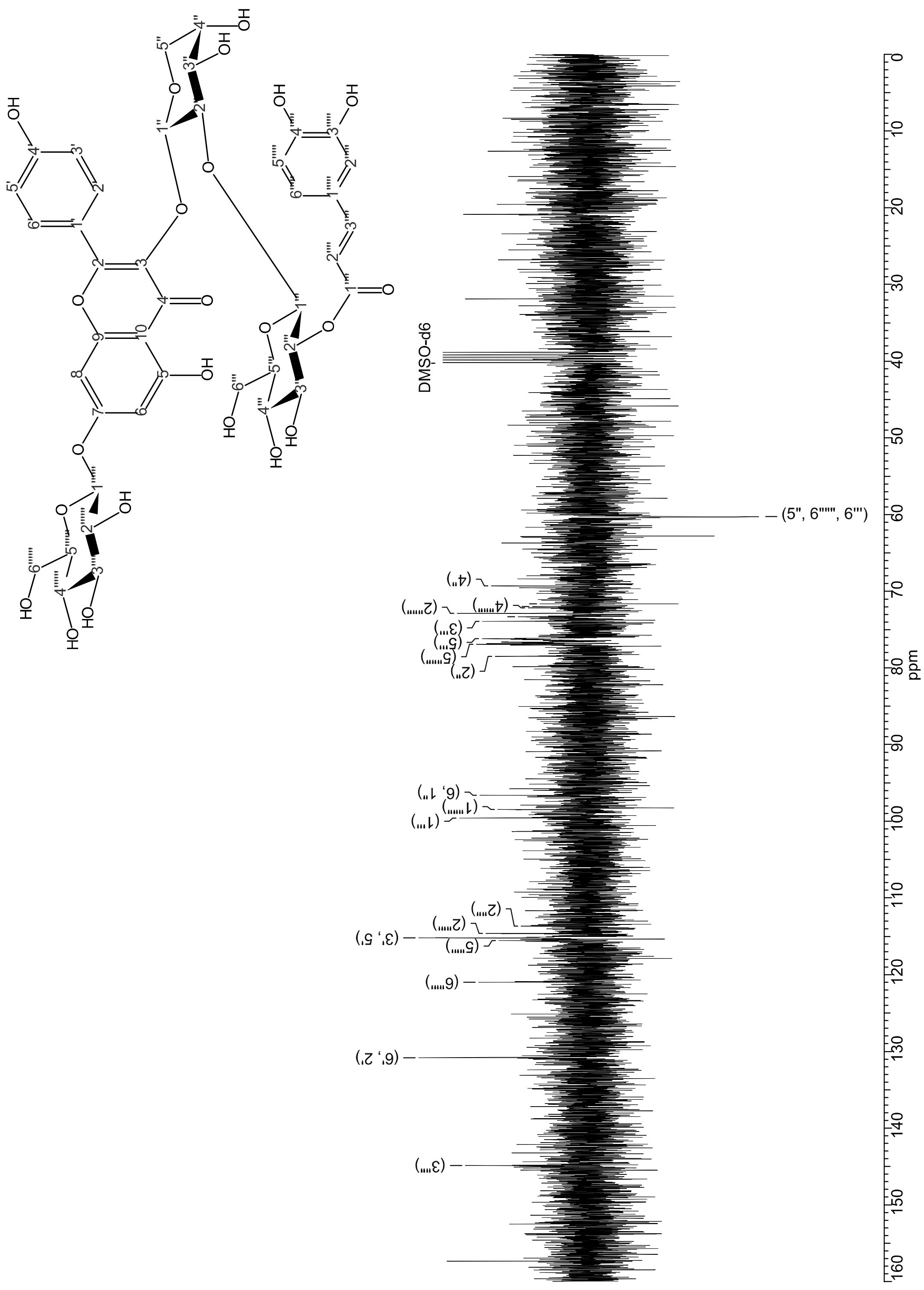

Figure S25. H-H-COSY of **4** (600 MHz, DMSO-d_6).

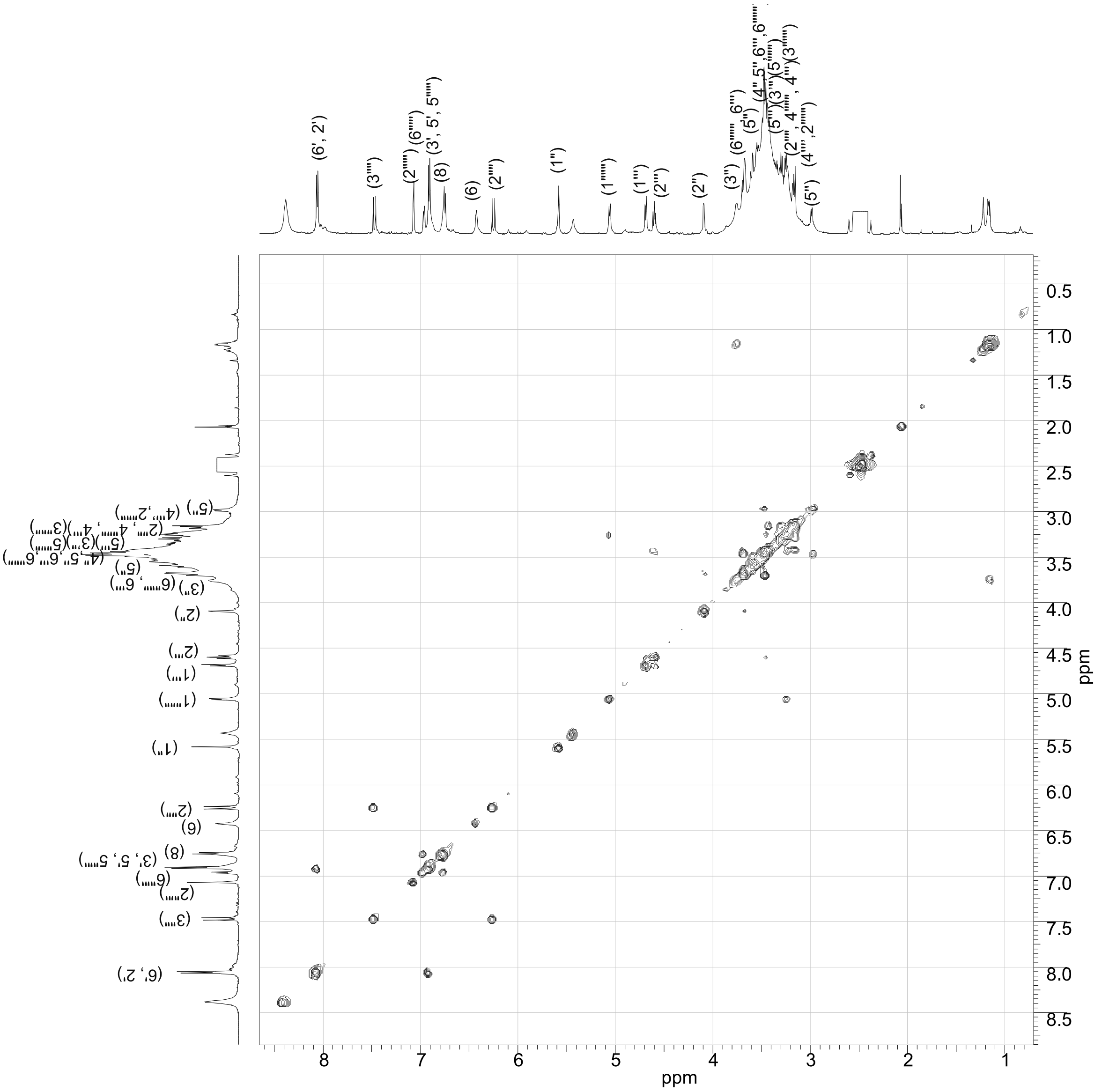

Figure S26. HSQC of **4** (600 MHz, DMSO-d_6).

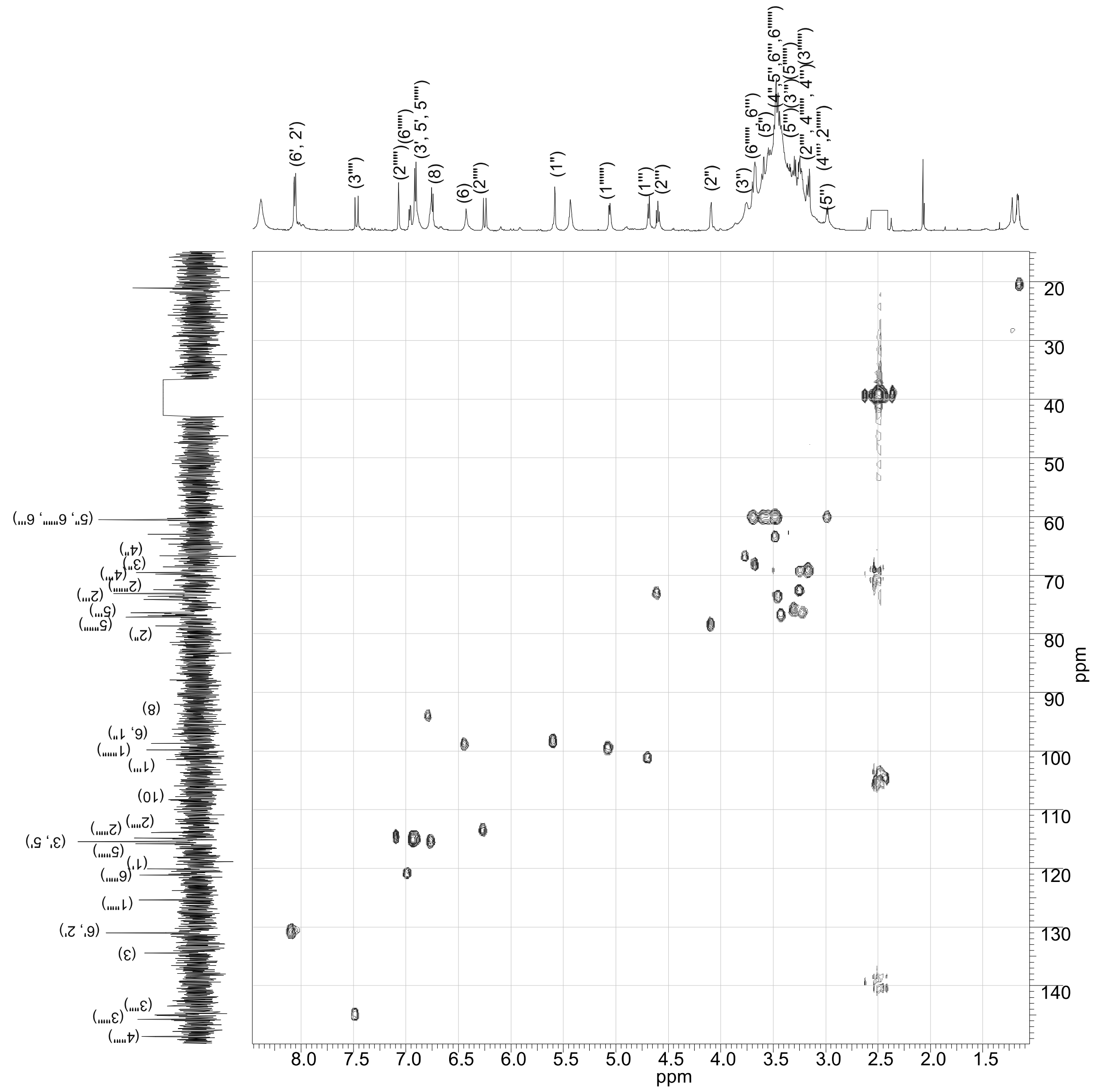

Figure S27. HMBC of **4** (600 MHz, DMSO-d_6).

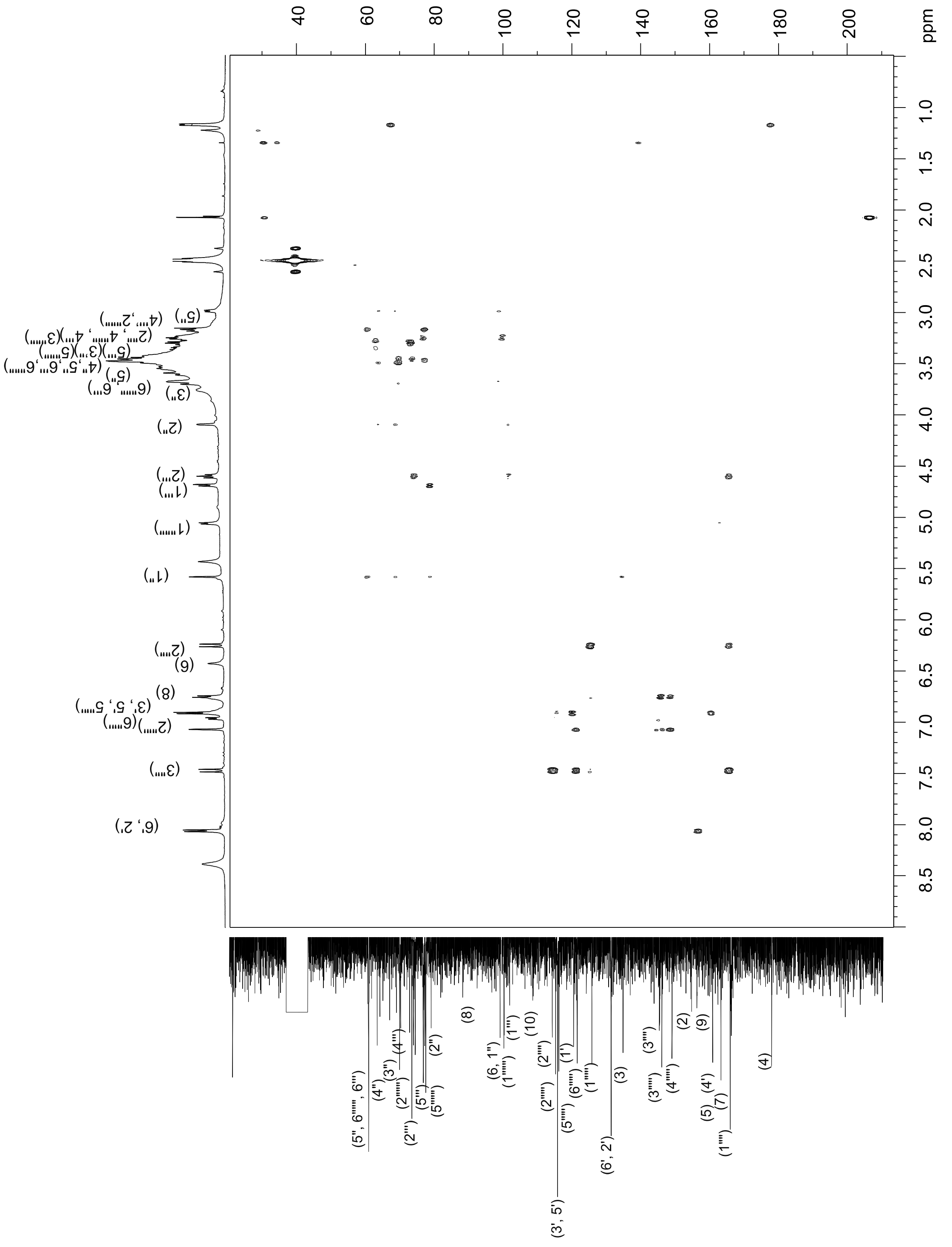

Supplement: Supplementary file 1 [file molecules-19-06727-s001.pdf]
